# Sputum DNA sequencing in cystic fibrosis: non-invasive access to the lung microbiome and to pathogen details

**DOI:** 10.1186/s40168-017-0234-1

**Published:** 2017-02-10

**Authors:** Rounak Feigelman, Christian R. Kahlert, Florent Baty, Frank Rassouli, Rebekka L. Kleiner, Philipp Kohler, Martin H. Brutsche, Christian von Mering

**Affiliations:** 1 Institute of Molecular Life Sciences, University of Zurich, Zurich, Switzerland; 2 Swiss Institute of Bioinformatics, Zurich, Switzerland; 3 Pneumology and Sleep Medicine, Cantonal Hospital St. Gallen, St. Gallen, Switzerland; 4 Infectious Diseases and Hospital Epidemiology, Children’s Hospital of Eastern Switzerland, St. Gallen, Switzerland; 5 Infectious Diseases and Hospital Epidemiology, Cantonal Hospital St. Gallen, St. Gallen, Switzerland

**Keywords:** WGS metagenomic sequencing, Cystic fibrosis, Sputum, COPD, Lung metagenome

## Abstract

**Background:**

Cystic fibrosis (CF) is a life-threatening genetic disorder, characterized by chronic microbial lung infections due to abnormally viscous mucus secretions within airways. The clinical management of CF typically involves regular respiratory-tract cultures in order to identify pathogens and to guide treatment. However, culture-based methods can miss atypical or slow-growing microbes. Furthermore, the isolated microbes are often not classified at the strain level due to limited taxonomic resolution.

**Results:**

Here, we show that untargeted metagenomic sequencing of sputum DNA can provide valuable information beyond the possibilities of culture-based diagnosis. We sequenced the sputum of six CF patients and eleven control samples (including healthy subjects and chronic obstructive pulmonary disease patients) without prior depletion of human DNA or cell size selection, thus obtaining the most unbiased and comprehensive characterization of CF respiratory tract microbes to date. We present detailed descriptions of the CF and healthy lung microbiome, reconstruct near complete pathogen genomes, and confirm that the CF lungs consistently exhibit reduced microbial diversity. Crucially, the obtained genomic sequences enabled a detailed identification of the exact pathogen strain types, when analyzed in conjunction with existing multi-locus sequence typing databases. We also detected putative pathogenicity islands and indicators of antibiotic resistance, in good agreement with independent clinical tests.

**Conclusions:**

Unbiased sputum metagenomics provides an in-depth profile of the lung pathogen microbiome, which is complementary to and more detailed than standard culture-based reporting. Furthermore, functional and taxonomic features of the dominant pathogens, including antibiotics resistances, can be deduced—supporting accurate and non-invasive clinical diagnosis.

**Electronic supplementary material:**

The online version of this article (doi:10.1186/s40168-017-0234-1) contains supplementary material, which is available to authorized users.

## Background

Cystic fibrosis (CF) is one of the most prevalent genetic disorders in the Caucasian population, affecting about one in 2500 newborns [1]. This autosomal recessive condition affects mostly secretory organs, such as the pancreas, liver, and lungs. CF is caused by mutations in the *Cystic Fibrosis Transmembrane conductance Regulator* (CFTR) gene, whose protein product is involved in the transport of chloride ions across the apical membrane of epithelial and blood cells. Loss of CFTR protein function causes thickened extracellular mucus to accumulate, which impairs mucociliary clearance in the airways [2]. CF prominently leads to microbial pathogen colonization in the lung, followed by recurrent pulmonary infection and chronic inflammation [3]. Treatment options exist, including mechanical and enzymatic mobilization of mucus, drug therapy to improve residual CFTR function [4], antibiotic therapy to reduce pathogen load, anti-inflammatory drugs, and lung transplantations. Nevertheless, for the majority of patients, the condition leads to progressive pulmonary damage and eventually respiratory failure and death.

The CF lungs are colonized by a number of pathogenic bacteria, commonly including *Staphylococcus aureus*, *Pseudomonas aeruginosa*, *Haemophilus influenzae*, and *Burkholderia cepacia* [5]. While prompt and aggressive antibiotic therapies can often control infections, prolonged antibiotic treatments may favor the emergence of antibiotic resistances and can facilitate colonization by multidrug-resistant pathogens such as *Achromobacter xylosoxidans* and *Stenotrophomonas maltophilia* [6, 7]. Currently, culture-based techniques are routinely employed to identify and classify lung pathogens, often using selective culture media designed for specific groups of pathogens [8]. However, the culture conditions and procedures are necessarily biased towards known, previously encountered pathogens—whereas novel, slow-growing or rare microbes might potentially be missed (e.g. atypical mycobacteria). Meanwhile, the taxonomic identification of observed pathogens often has limited resolution, and the physiology and resistance profiles of the colonies are not backed up using genomic information. Lastly, the “background” community—opportunistic or accidental members of the lung microbiome—is not routinely studied for clinical use [9, 10], despite its potential to harbor antibiotic resistance genes and to elicit or modulate immune responses.

Culture-independent, genomic sequencing techniques offer potential alternatives for identifying pathogens and opportunistic colonizers and for guiding therapeutic decision-making. However, such methods are not routinely applied for CF management. In research settings at least, what has been used most frequently are PCR-based surveys of the 16S ribosomal RNA gene [11, 12], which are however of limited taxonomic and functional resolution.

Here, we develop a pragmatic approach that aims to maximize molecular information, while minimizing patient discomfort and risk exposure. To achieve this, we sequence DNA from non-invasive sputum samples, without prior removal of host DNA and without complex enrichment or depletion protocols. Forgoing host DNA depletion yields a substantial fraction of sequence reads that are of human origin, and there will be contributions from the upper respiratory tract and oral cavity [13], but the simplicity of the approach has the unique advantage of providing an unbiased, comprehensive, and reproducible set of reads from the lower respiratory tract as well. There have been only few studies so far that took a somewhat similar approach [14–18], each with slight differences in sample processing, sequence analysis, and focus.

For the current study, we collected and sequenced sputum samples from the following categories: adult and pediatric CF patients (6 samples), chronic obstructive pulmonary disease (COPD) patients (4 samples), and healthy individuals (7 samples). Using whole-genome shotgun sequencing, we observed several known pulmonary pathogens whose genome coverage routinely exceeded 95%, allowing us to type the strains with very high precision using existing multi-locus sequence typing (MLST) databases. Patient-specific differences from reference strains are noted and discussed. Using the workflow we established, a detailed genomic profile can be generated for any lung pathogen from which sufficient DNA can be prepared, including also potentially unknown pathogens.

## Results and discussion

### Sputum samples contain diagnostically useful DNA

**Fig. 1:**
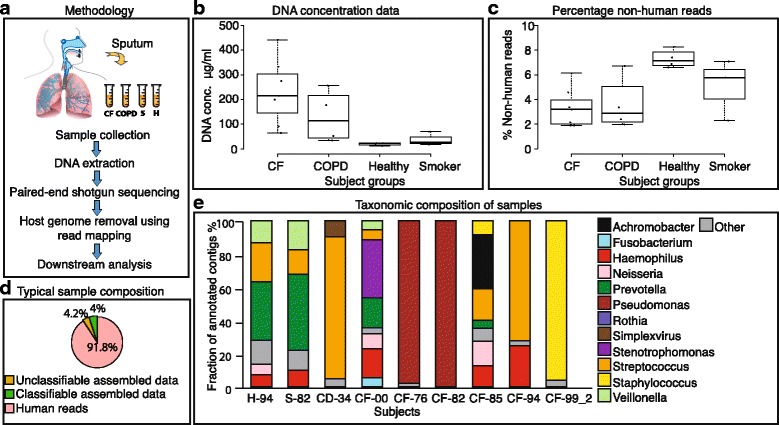
Sputum metagenomics workflow. **a** Overview of the procedure. **b** Concentration of extractable DNA in sputum, across subject groups. **c** Fraction of non-human DNA sequence reads across subject groups. **d** Fraction of DNA sequence reads of a representative healthy sample, further broken down according to taxonomic assignability to the assembled nucleotides from non-human fraction. **e** Taxonomic composition of all taxonomically assignable, non-human sequences, at genus level (for each of the control groups, only one representative sample is shown). All genera constituting at least 4.5% of the annotated fraction in each sample are assigned with a color code

We collected sputum samples from a total of 17 subjects: 6 CF patients, 4 patients with COPD, and 7 healthy controls (3 smokers, 4 non-smokers). CF patients were able to expectorate sputum spontaneously; for others, induction by hypertonic saline inhalation was used. The sample amounts, dates, and pulmonary function parameters are summarized in Additional file 1: Table S4. We extracted the total DNA from each sputum sample and sequenced it without removal of the human DNA, using the Illumina HiSeq 2000 platform (Fig. 1a). In total, the average sequencing depth was around 30 million read pairs per sample (Additional file 2: Figure S1). We then separated and quantified human and non-human read pairs; the latter were further assembled into contigs (Additional file 3: Table S1) and annotated using homology-based searches (see “Methods”).

The observed DNA concentrations in sputum varied across subject groups, with CF and COPD patients presenting on average higher DNA concentrations than the healthy controls (Fig. 1b). In all samples, a large fraction of sequenced DNA (>90%) was of human origin (Fig. 1c). This agrees with previous studies, which suggested that free human DNA in the lung stems from disintegrating inflammatory cells such as neutrophils that release DNA into sputum, particularly in the case of the diseased lung tissue, leading to an increased sputum viscosity [19, 20]. Conversely, healthy subjects produced smaller volumes of sputum with lower DNA concentrations, but their residual DNA was also mostly of human origin (Additional file 2: Figure S2).

### Lung microbiota composition varies strongly across individuals

After a limited (conservative) assembly of the non-human sequence fraction into contigs, approximately half of the assembled nucleotides could be assigned a taxonomic identity through homology searches, irrespective of subject groups (Fig. 1d). For healthy subjects, we found an overall higher diversity (average Shannon entropy of 3.07 in the healthy non-smoker lungs, versus 1.08 in the CF lungs, *p* < 0.038). Interestingly, the diversity was found to be reduced not only in the sputum of CF and COPD patients but also to some extent in smokers (Additional file 2: Figure S3). The most abundant taxa in healthy subjects were *Prevotella*, *Streptococcus*, *Veilonella*, *Haemophilus*, and *Neisseria* (Fig. 1e). Previous studies have indicated that the healthy lung does not harbor a stable and specific microbiome, but rather a mixture of microbes from the upper respiratory tract and oral cavity [21–24]. In agreement, many of the microbes we identified in healthy subjects corresponded to known oral (and occasionally also gastrointestinal or vaginal) flora.

In contrast, the microbiome composition in CF subjects was highly variable and distinct from standard oral microbiomes. Each patient harbored a unique community, often dominated by one or a few principle colonizers/pathogens such as *Pseudomonas*, *Staphylococcus*, *Stenotrophomonas*, or *Achromobacter*. (Fig. 1e, Additional file 2: Figure S4). Compositionally, the CF flora only marginally overlapped with those of the healthy and smoker populations.

### Observed microbiota in CF subjects match clinical diagnosis

For five of the six CF subjects, all bacterial pathogens identified in the clinical culture diagnosis were also identified through the DNA sequencing, each with at least 10,000 bp mapped to their genome (Additional file 4: Table S2). For example, we confirmed the presence of the typical CF pathogens *Achromobacter* and *Staphylococcus* in the sample CF-85 (for all subjects, see Additional file 5: Table S3). In this particular patient, we also observed multiple instances of antibiotic resistance genes, including genes annotated to potentially diminish effectiveness of the ongoing antibiotic treatment (see below, “Prediction of antibiotic resistances and fitness-conferring mutations”). We also observed *Prevotella*, *Neisseria*, *Streptococcus*, *Haemophilus*, and others in lower abundances. These latter genera are typically viewed as commensals, but it cannot be ruled out that they contribute to pathogenesis as well. In pediatric CF patient CF-00, we detected *Pseudomonas*, a multidrug-resistant (MDR) *Stenotrophomonas*, and several other common microbial inhabitants. In contrast, the microbiomes of adult CF patients CF-82 and CF-76 were primarily dominated each by a single pathogen, *Pseudomonas*, with very low relative abundances of others, such as *Streptococcus*. Here and in similar “consolidated” situations, competition between microbes may have suppressed diversity [25].

Patients CF-99 and CF-94 exhibited a microbial community largely dominated by *Staphylococcus* and *Streptococcus*, respectively. These were two of the patients for which antibiotic treatment was ongoing; use of antibiotics is thought to correlate inversely with diversity and to instigate significant changes in bacterial community structure, especially in younger patients that harbor a relatively rich and susceptible microbial community as compared to older patients that often develop resilient communities [26–30]. Hence, in patients CF-94 and CF-99, lack of a diverse microbial community is consistent with the ongoing treatment at the time of sample collection (Additional file 1: Table S4 and Additional file 3: Table S5); in this case, no resistances against the administered antibiotics had been detected in clinical testing.

### The samples from COPD patients reveal intermediate microbial complexity

We analyzed the microbiome diversity for each COPD patient and found that, of the four samples collected, CD-47 had the largest and most diverse population, closely resembling the composition of a healthy microbiome. Subject CD-34 was unique in that it was the sole sample in which we detected significant amounts of a virus, *Herpes simplex virus*. This virus was covered deeply enough to be partially assembled and was seen against a background of *Streptococcus*, *Rothia*, and *Haemophilus*, with *Fusobacterium* and *Prevotella* greatly reduced (Additional file 2: Figure S4). Overall, the samples from COPD patients were somewhat more difficult to characterize. On several occasions, we failed to detect eukaryotic genera that had been observed in the clinical culture-based diagnostics. For example, in patient CD-42, we were able to reliably detect the presence of several bacterial community members (confirmed by clinical microscopy results) but were unable to confirm the presence of *Candida*. Since eukaryotic genomes tend to be larger and more challenging to assemble, they may sometimes fail to be detected in sufficient numbers in our approach.

### Entropy landscapes allow the detection of clonally expanded pathogens

**Fig. 2:**
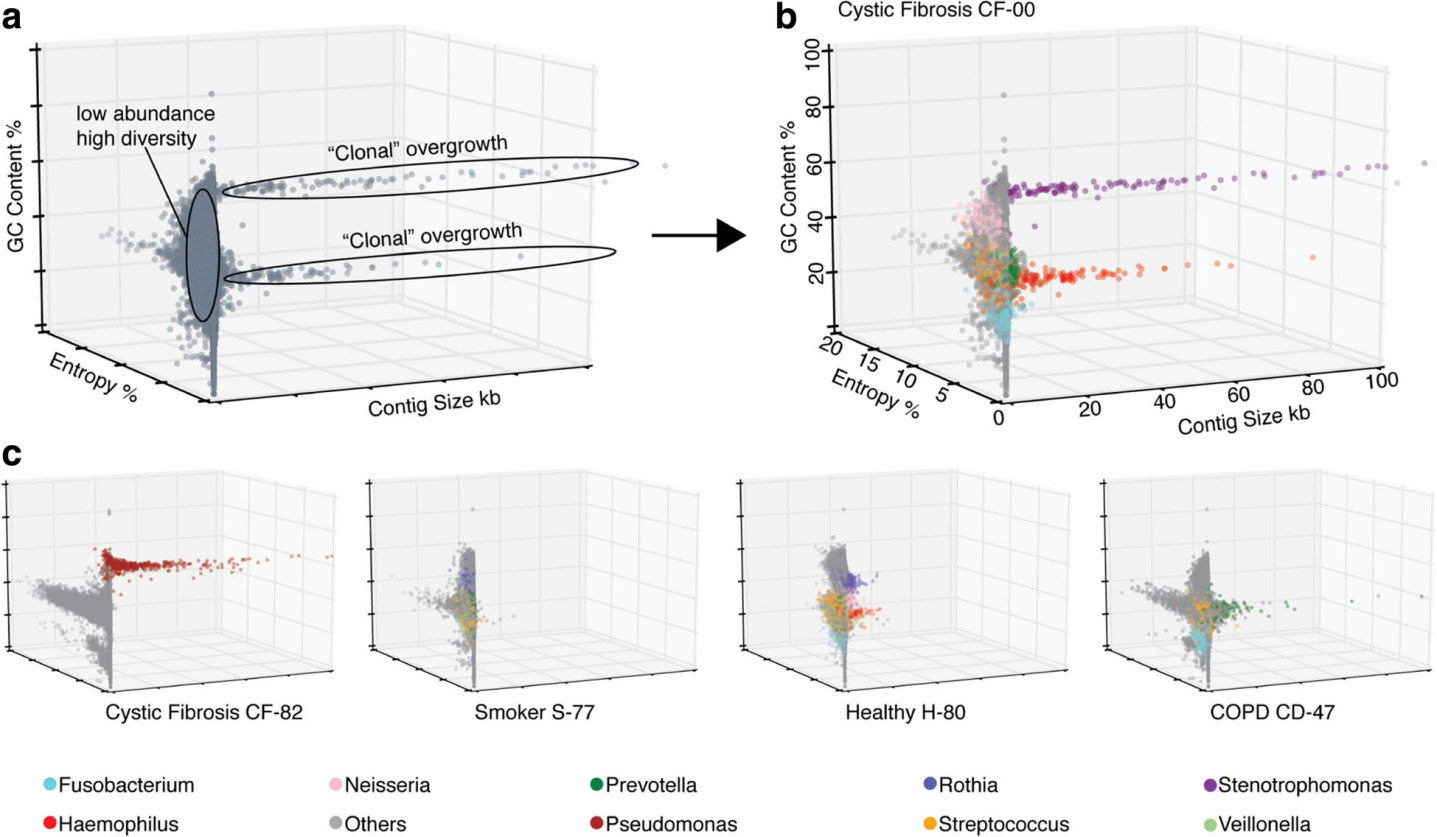
Pathogen overgrowth can be separated from background diversity. Sequence contig feature plots (“entropy landscapes”), depicting at least one sample from every subject group. Each data point represents an assembled contig, with colors corresponding to genus level taxonomy annotations. The three axes show contig length (X-axis), contig sequence heterogeneity (entropy, Y-axis), and GC-content (Z-axis). **a** Magnified view of the plot of patient CF-00 without taxonomic annotation. **b** The same plot (CF-00) but with taxonomic annotation. **c** Representative plots of one subject from each group. Throughout, genera constituting less than 5% of the annotated fraction, as well as unannotated contigs are shown in *gray color*

The microbial community in the sputum of CF patients is expected to be heavily skewed—a small number of entrenched, chronic pathogens stand out against a more diverse background of contaminants and putatively harmless colonizers [31, 32]. We devised a three-dimensional binning strategy adapted to this situation, in which each contig is assessed in terms of (i) GC-content—as a proxy for broad taxonomic identity, (ii) length—as a proxy for assembly depth, and (iii) sequence homogeneity within the assembly—as a proxy for clonality. The latter measure is expressed as entropy, where a small entropy value reflects a low number of mismatched sites in the assembly of a given contig. Low-entropy contigs should reflect clonal or near-clonal microbial strains (within the limits set by sequencing accuracy and depth). We used these three measures to visualize the entire non-human sequencing result of any patient of interest in a three-dimensional binning plot (Fig. 2). For those contigs whose taxonomic identity could be confidently inferred, we additionally used a color code to highlight groups of sequences that might putatively belong to the same genus.

In Fig. 2, entropy landscapes are used to visualize the lung community composition of two CF patients with distinct dominant pathogens, as well as one representative sample each from COPD, smoker, and non-smoker groups. For CF-00, the plot shows two likely clonal overgrowths with distinct GC content (Fig. 2a), suggesting chronic infections by two distinct pathogen species. Indeed, annotation revealed these contig groups to consist exclusively of members of the genera *Stenotrophomonas* and *Haemophilus*, respectively (Fig. 2b).

These observed landscapes are characteristic of microbial communities with one or a few dominant members that have grown clonally to occupy a sizeable proportion of their niche. In contrast, the healthy and smoker groups generally were not dominated by few clonal species, as reflected in the absence of clustered low-entropy contigs (see Additional file 2: Figure S5 for plots of each subject sampled).

The entropy landscapes allow the visual separation of likely oral contaminants and low-abundance colonizers from the clonal pathogen(s) growing chronically. Furthermore, any non-annotated contigs that visually cluster within the pathogen contigs may indicate undocumented genomic regions, which would have been recently introduced into the pathogen genome and may not be known from reference strains in databases. Hypothetically, even atypical pathogens that are not yet annotated in any database would become discernable, although we have not encountered such a case among our samples.

### Multi-locus sequence typing of pathogens using unbiased sputum sequences

Multi-locus sequence typing (MLST) is a well-established method to characterize isolates of a given microbial species in the context of previously observed strains of that species, via DNA sequencing of a limited, pre-defined group of diagnostic genes [33]. Traditional MLST requires the isolation and culture of microbes of interest, followed by specific PCR assays targeting the genes used for strain typing. In contrast, by using WGS sequencing data, we omit these steps and directly proceed to characterizing strains of interest from the mixture. Importantly, there is no need to decide, ahead of the experiment, which strains are to be typed since no specific PCR is required. As long as a species or genus has been previously subjected to MLST genotyping (i.e., a well-populated MLST database with corresponding marker genes is available), it can be characterized. In the following, we describe two examples of MLST strain genotyping in the sputum of CF patients—one yielding a previously observed strain from a well-sampled strain collection, the other yielding a more exotic strain for which even the exact species designation remains unclear (it may belong to a new, as yet unnamed species).

**Fig. 3:**
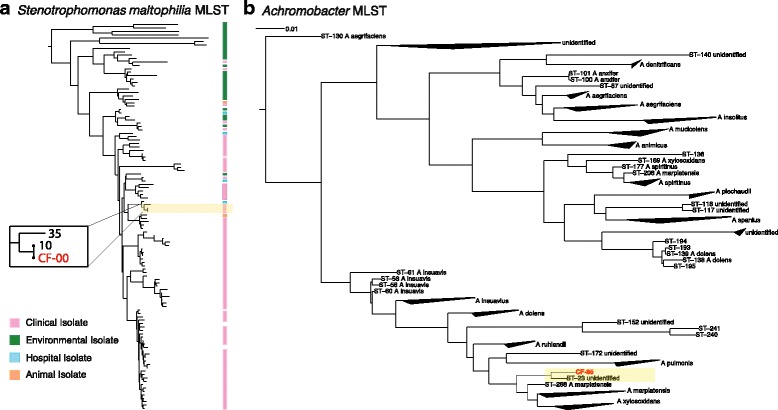
High-precision strain typing from sputum sequences. Multi-locus sequence typing for two selected pathogen strains from CF samples. *Yellow color* highlights the phylogenetic position of the strains observed in this study, relative to previously typed strains deposited in MLST databases. **a** Patient CF-00 is colonized by a *S. maltophilia* strain that has close relatives in the database. Isolation sources of database strains are shown color-coded. **b** Patient CF-85 has a strain from the genus *Achromobacter*, for which no close relatives have been observed before (the strain likely does not belong to a named species). All monophyletic clades with 95% members from a single species have been collapsed

We characterized a strain of *S. maltophilia* which we had observed in the sputum of patient CF-00. *S. maltophilia* is an intrinsically multidrug-resistant (MDR) opportunistic pathogen that has been isolated from several water-associated environments inside and outside of hospital premises [34]. Like many free-living opportunistic pathogens, it possesses a large and versatile genome that allows it to colonize diverse environments and degrade toxic compounds such as antibiotics, even using them as food sources [35]. *S. maltophilia* exhibits high levels of genetic diversity, making it hard to precisely track the source of infections and distribution of isolates in hospitals. We performed MLST using a standard set of seven housekeeping genes [36], all of which were found with 100% sequence coverage in our sample. We observed that the patient harbored a single strain (likely from a single infection event), which was 100% identical to a strain encountered previously, in a CF case in the UK. We placed this strain, together with other strains observed previously, in a phylogenetic tree constructed from the MLST alignment, see (Fig. 3a). The tree was then annotated with the sampling origin of each strain: clinical, environmental, hospital environment, or animal-associated. The phylogenetic analysis revealed a clear clustering of clinically obtained strains in a single clade, suggestive of specialization, and frequent transfers from patient to patient. Overall, we found this genome to be very well recovered from the sputum, with an analysis using CheckM [37] reporting it to be 97.2% complete.

Next, we observed and characterized a putative *Achromobacter* strain from patient CF-85. Members of the *Achromobacter* genus form a group of gram negative, strictly aerobic, motile bacteria of which more than 10 species are currently known. The majority of strains isolated from CF patients belong to the species *A. xylosoxidans*, which is also an intrinsically multidrug-resistant opportunistic pathogen [38]. Aside from the patients, strains can also be found in a variety of aquatic environments ranging from moist soils to dialysis solutions [39]. *Achromobacter* infections have been generally observed in older patients with pulmonary diseases, but their implication in deteriorating lung function has remained unclear [40, 41]. Accurate identification and discrimination of different *Achromobacter* species has been a challenging task due to limited taxonomic delineation. A recent study revealed that several commercial test systems used in different diagnostic laboratories were unable to distinguish different *Achromobacter* species infecting CF patients and would often identify them incorrectly as *A. xylosoxidans* [42].

Using the appropriate gene set for this genus, we again performed MLST. In this case, we did not find any matches to previously documented strains. Instead, we placed our sequences on a phylogenetic tree encompassing the entire genus, constructed using concatenated housekeeping genes from all species and type strains available in the PubMLST database (http://pubmlst.org/achromobacter/) [43] (Fig. 3b and see “Methods”). Our observed strain did not cluster with other identified strains, with the exception of a single unnamed and uncharacterized isolate from another CF case. The closest neighbors of these two strains in the tree were *A. marplatensis* and *A. pulmonis*. Although routine clinical analysis using microscopy had identified the strain as *A. xylosoxidans*, our phylogenetic analysis suggested otherwise; the two sequences were sufficiently removed from *A. xylosoxidans* to suggest a novel but previously unidentified clade. Independent studies [44] have also provided evidence to support the presence of as-yet unnamed and uncharacterized species in the *Achromobacter* genus, responsible for CF infections in patients. Further species divisions in this clinically relevant but undersampled genus are needed, and patient-derived genomes such as ours might provide valuable context.

### Prediction of antibiotic resistances and fitness-conferring mutations

Chronic colonizers can adapt to their host environment to sustain themselves under varying selection pressures, such as antibiotic treatments or the presence of potentially competing co-infections. This is particularly problematic in the case of infections caused by antibiotic-resistant bacteria in CF patients. For example, a five-fold increase in MRSA infections has been observed in the past 15 years [45], and 18.1% of *P. aeruginosa* infections in a population of primarily young adult CF patients were reported to be MDR [45]. These pathogens acquire various resistance mechanisms to therapeutic agents including altered membrane permeability, efflux pumps, or induced enzymatic modifications. Isolates with identical resistance patterns sometimes exhibit different genetic modifications, indicating that these bacteria can use distinct strategies to respond to similar environmental pressures [46].

**Fig. 4:**
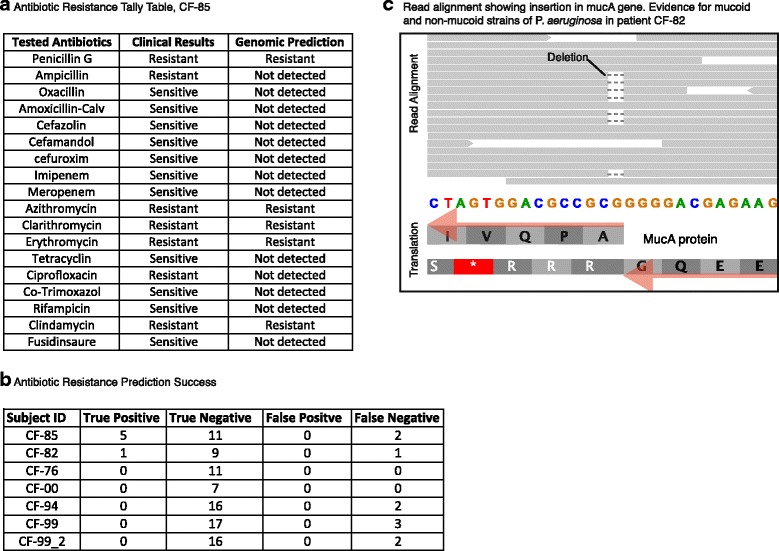
Prediction of antibiotic resistances and other phenotypes. **a** The *Achromobacter* strain isolated from patient CF-85 underwent routine clinical testing for antibiotic sensitivity; the compounds tested and the observed results are shown. This is contrasted with automated predictions based on the gene content of the sputum sequence data. **b** Summary table for all CF subjects, indicating the overlap between the resistance predictions and the clinical test results. **c** Read alignment against a section of the *mucA* gene from *P. aeruginosa*, from patient CF-82. Eleven reads show a wild-type sequence at this position, but 7 reads show a deletion event predicting a non-functional protein and a corresponding shift from a non-mucoid to a mucoid phenotype in this strain

To test whether sputum sequencing might help to guide antibiotic treatment, we screened for the presence of putative resistance-conferring genes and alleles. We compared all predicted open reading frames in each sample against a database of known and annotated antibiotic resistance genes (see Methods), employing conservative similarity cutoffs. For example, in the sputum of CF-85, we identified genes encoding for class A beta lactamases, which are classified as serine enzymes conferring resistance to penicillin. In the same sample, we also identified a 23S ribosomal RNA methyltransferase, conferring varying degrees of resistance to macrolide, lincosamide, and streptogramin B antibiotics (see Fig. 4a and Additional file 3: Table S5).

The predictions were validated independently by clinical resistance reports, where the majority of both “resistant” as well as “non-resistant” calls were confirmed (Fig. 4b). False predictions were limited to false negatives, i.e., clinically observed resistances which were not predicted based on sequence analysis. Interestingly, we additionally observed a virulence gene, mprF, annotated as providing resistance against naturally secreted antimicrobial peptides known as defensins. These cationic peptides are largely secreted by neutrophils and by the airway epithelium in the CF lungs to protect the epithelia against infections. *S. aureus* strains with resistance to defensins show a greater pathogenic potential [47]. Thus, the presence of such virulence factors is of general relevance to clinicians when designing treatments for CF patients.

Apart from antibiotic resistances, other phenotypes such as biofilm formation or exo-polysaccharide secretion may also be actively adapting in chronic colonizers. Indeed, it is known that chronic colonizers develop considerable genomic heterogeneity, which is perhaps maintained by specialization or balanced selection [48, 49]. We conducted a systematic search for genomic heterogeneity using the FreeBayes tool and observed varying levels of sequence variation (see Additional file 6: Table S7 and “Methods”). With our genomic read coverage often below 10×, we are somewhat limited in power, but a conservative search shows that two of the pathogens are nearly clonal, with less than 20 variants observed for each (*Staphylococcus* in patient CF-85 and *Pseudomonas* in patient CF-76). In contrast, the remaining nine pathogens tested show considerable heterogeneity, with hundreds of sequence variants observed in each strain (Additional file 6: Table S7). In the case of *P. aeruginosa*, this kind of sequence heterogeneity has been particularly well studied, and a set of 52 genes has been shown to be likely adaptive inside the lung [50]. Of these, we indeed found five with SNPs in one of our patients, CF-82. As an example, we highlight the *mucA* gene, which controls the mucoid/non-mucoid phenotype. Initial isolates of *P. aeruginosa* from CF patients are generally non-mucoid and responsive to antibiotics. However, during protracted infections, these pathogens start overproducing an exo-polysaccharide known as alginate which is a polymer of α-D-manuronic acid and L-glucuronic acid [51], eventually leading to their transition into a mucoid phenotype. Mucoid *P. aeruginosa* is immune to several antibiotics and to phagocytosis [52]. Correspondingly, the mucoid phenotype is directly linked with poor clinical outcome for patients. According to the clinical laboratory report, the sputum of CF-82 harbored both mucoid and non-mucoid *P. aeruginosa* strains. To better understand the underlying genetic modifications that lead to this phenotypic transition, we inspected the *mucA* gene, which encodes for a trans-membrane σ-factor responsible for limiting expression of the 12-gene alginate operon (*algA-algD*). Loss of function mutations in the *mucA* gene typically result in production of alginate, in turn giving rise to a mucoid phenotype. In CF-82, we indeed identified 7 sequence reads showing a single-nucleotide deletion at position 429 in the *mucA* gene (Fig. 4c), leading to a truncated and presumably non-functional protein. In contrast, 11 reads supported the presence of a non-mutated, fully functional protein; in combination, these reads confirm and explain the clinical observation.

### Genome comparisons reveal patient-specific pathogen features

**Fig. 5:**
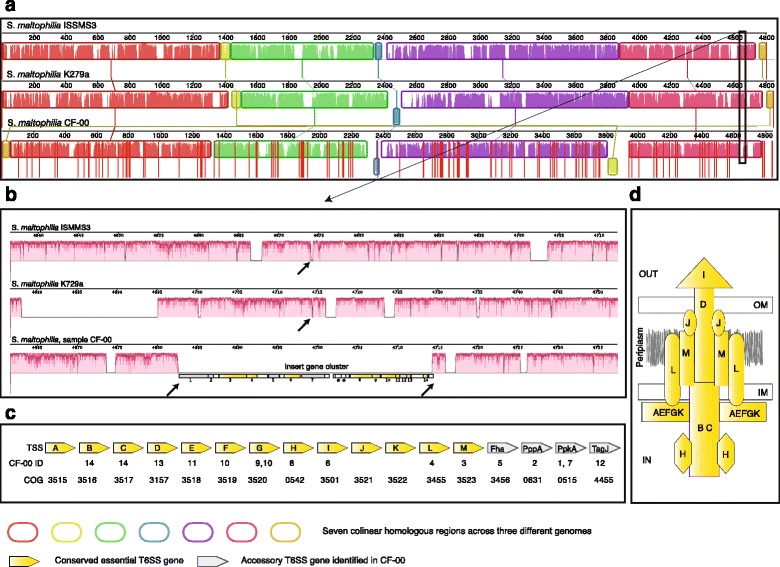
Pathogen genome comparisons reveal patient-specific additions. Two public reference genomes of *S. maltophilia* are compared against assembled contigs from patient CF-00. **a** Genome-wide alignment showing blocks of colinearity, additions, and deletions. *White stretches* indicate unalignable, unique regions in each genome. *Vertical red lines* separate individual assembled contigs. **b** Magnified view centered on a genomic region that is unique to the strain in patient CF-00. Genes with homology to type six secretion system (T6SS) have been labeled with numerical IDs (see panel **c** below). Genes marked with an *asterisk* showed no detectable homology in sequence databases. **c** The core gene cluster of T6SS is depicted in *yellow*; additional accessory T6SS genes which are also observed in patient CF-00 are colored in *gray*. **d** Schematic model of the T6SS protein structure based on present knowledge

The MLST procedure allowed us to precisely characterize the taxonomic identity of strains of interest but provided little phenotypic information regarding pathogenicity or metabolic characteristics. Moreover, this information is not routinely available from culture-dependent techniques in the clinic. To address this, we collected all contigs assembled from CF-00 belonging to *S. maltophilia* and aligned them against two closely related clinical database strains, Sm K279a and Sm ISSMS3 [GenBank accession NC_010943 and NZ_CP011010, respectively] (Additional file 2: Figure S6). This identified seven large-scale homologous regions (see Fig. 5a). These regions were interspersed by non-homologous intervals unique to each genome, including some in the assembled genome from patient CF-00. The assembled genome also exhibited some genomic rearrangements with respect to the reference strains.

We next made gene predictions for all the unique genomic regions of size 1 kb or greater present in the reconstructed genome. Interestingly, we found a large region of 23 genes (Fig. 5b), 14 of which were found to code for a virulence-associated type VI secretion system (T6SS). T6SS systems typically consist of a conserved cluster of 13 core genes, 10 of which were observed in our gene set together with 4 additional non-conserved T6SS accessory genes (Fig. 5c) responsible for post translational regulation based on orthology predictions. T6SS secretion systems (Fig. 5d) were first described a decade ago in *Vibrio cholerae* [53] and since then have been studied in several gram-negative bacteria, including *P. aeruginosa* [54]. They allow the secretion of a range of substrates such as toxins, adhesins, hydrolytic enzymes, and effector proteins and have been classified into four subtypes [55]. T6SS have been associated with biofilm formation and antagonistic or bactericidal functions towards competing bacterial species [54]. They are frequently observed in genomic islands, and their presence shows little correlation to bacterial taxonomy, suggesting that they are frequently acquired through horizontal gene transfer [56].

In the past decade, T6SS have repeatedly been reported to be absent in all *S. maltophilia* isolates, both in clinical and environmental isolates [57–59]. Its presence in our clinical isolate thus highlights its outstanding ability to adapt to new hosts and surroundings. In addition to our observation, recent observations in new *S. maltophilia* strains [60] were also supportive of T6SS systems (although the genomes are yet to be published).

The spread of such virulent secretory systems in multidrug-resistant CF pathogens provides a unique opportunity to identify new targets for designing innovative treatments. Anti-virulence drugs [61] that target specific secretion systems to disarm the bacteria can be a likely alternative to conventional antibiotics. Thus, whole genome reconstruction of highly abundant bacteria in the patient samples provides an exclusive possibility to study idiosyncratic genomic regions and to identify potential drug targets for targeted treatments.

## Conclusions

In this study, we have introduced a culture-independent technique for characterizing airway pathogens in the chronically inflamed lung, using routine non-invasive sputum sampling coupled to unbiased WGS sequencing. We chose not to subject the sputum samples to any particle-size selection or human host DNA depletion, although protocols specifically suitable for sputum samples have been developed [16, 62]. This means that our sequence reads will include not only a large number of human genome-derived sequences but also extracellular DNA from microbial cells that might no longer be viable. On the other hand, the simplified processing means that experimental biases are minimized, and most DNA-containing pathogens should be accessible (particularly given the ongoing increases in sequencing throughput). Our approach should provide valuable information complementing routine culture-based clinical microbiology results in the future. This could help to design tailored treatment regimes by reducing the risk of ineffective treatment.

We find, firstly, that WGS sequencing can serve to describe the broad taxonomic composition of the lung microbiome, particularly when combined with reference databases. Reference information is still incomplete and can bias the results, but for human-associated microbes, it is growing at a remarkable pace [63, 64] and should make WGS-based taxonomic classification ever more accurate in the future.

Secondly, various binning approaches [65] can be used to partially assemble genomes of interest from the WGS data. In the case of chronic infections originating from one or a few clonal invasions, we find that contig-by-contig entropy is a good measure for isolating pathogens from contaminants and from the complex colonizing background of microbes. Such an approach may help clinicians to more precisely identify the disease-causing pathogen.

Thirdly, for those pathogens that have already been well studied in molecular terms, we demonstrated that MLST can be applied directly using WGS data. This allows the exact strain identity to be established, leveraging the power of MLST databases and cataloged strain observations. Importantly, this can significantly simplify the process of tracing infection outbreaks at clinics using untargeted, retrospective data.

Lastly, the partially assembled genomes and the remaining unassembled contigs could be informative with regard to the expected efficacy of treatment options. Antibiotics susceptibility testing is an important part of clinical management, although its efficacy has been questioned [66, 67]. We find that antibiotic resistances can be predicted from the metagenome, but while this is substantially supported by confirmatory clinical tests, it is not yet entirely error-free. This is likely to improve with better data curation in the resistance databases, but the most challenging resistances to predict correctly will be those that arise from specific mutations in normal, cellular genes [68]. Importantly, aside from predicting resistances, WGS data may guide the decision as to which antibiotics to include in clinical testing in the first place, particularly for the second-generation antibiotics designed to counteract or circumvent known resistances.

Overall, WGS sequencing of sputum may become one of the building blocks supporting the advancement of a more personalized medicine. It yields not only deep insights into the lung microbiome by allowing an unbiased metagenomic dissection of microbial pathogens but also enables analysis of human genomic DNA for host genotyping (e.g., for host susceptibility to infection or for unexpected treatment responses). For routine tracking, deep WGS sequencing could be alternated with more shallow survey sequencing or 16S sequencing; the latter are likely sufficient to quantify changes in community composition, with deep sequencing only necessary when the new pathogens invade.

## Methods

### Sputum samples and DNA extraction

Sputum was either produced spontaneously (in the case of CF and COPD patients) or after induction by hypertonic saline nebulization (in the case of healthy control subjects). The sampling was conducted at the Cantonal Hospital St. Gallen and at the Children’s Hospital of Eastern Switzerland. Healthy control subjects were free of symptoms of respiratory discomfort and did not show overt infections. All study participants provided informed consent. The study was approved by the Cantonal Ethics Committee, St. Gallen (EKSG 13/112). The sputum samples were weighed and aliquoted into sterile tubes. After dilution in Sputolysin (Calbiochem Corp., San Diego, CA, USA), total DNA was extracted using the High Pure PCR Template Preparation Kit (Roche, Basel, Switzerland) according to manufacturer’s instructions. DNA concentration was measured using the ACTgene UV99 spectro-photometer at a wavelength of 260 nm, and samples were stored at −20 °C. As the starting material was not limiting, and sufficient amounts of DNA were available, no extra amplification step was deemed necessary and no extraction blanks for PCR/sequencing contamination control were processed.

### Whole-genome shotgun sequencing

The TruSeq DNA Sample Prep Kit v2 (Illumina Inc., California, USA) was used for library generation. The quality and quantity of the enriched libraries were validated using a Qubit® (1.0) fluorometer and the Caliper GX LabChip® GX (Caliper Life Sciences, Inc., USA). The libraries were normalized to 10 nM in Tris-Cl 10 mM, pH 8.5 with 0.1% Tween 20. The TruSeq PE Cluster Kit v3-cBot-HS (Illumina, Inc., California, USA) was used for cluster generation using 2 pM of pooled normalized libraries on the cBOT. Paired-end sequencing was performed on the Illumina HiSeq 2000 at 2 X101 bp using the TruSeq SBS Kit v3-HS (Illumina, Inc., California, USA). Reads were quality-checked with FastQC [69].

### Removal of the host genome and assembly

We used Bowtie2 [70] to align the paired-end reads against the human reference genome, assembly Hg19 [71]. Read pairs were omitted from any further downstream analysis if one or both mates from the pair aligned to the human genome. We assembled contigs from the remaining read pairs using the SPAdes assembler [72] under the “only assembly” setting.

### Taxonomic annotation and diversity estimation

We searched the contigs against the NCBI nucleotide database (as of June 2014) using Blastn [73] with an *e* value cutoff of *e* ^−15^. The most common recent ancestor of all genomic sequences that aligned to a given contig with a bit score within 10% range of the highest scoring alignment was used to taxonomically annotate the contig. This procedure largely excluded the analysis of phages and viruses because these tend to be poorly represented in databases and taxonomies. We independently cross-checked our microbial assignments by running MiDAS, a strain-level taxonomy analysis tool based on a large collection human-associated microbial pathogens [74]. As shown in Additional file 7: Table S6, there is a good agreement between both approaches for the more abundant genera, while some low-abundant genera are missed by MiDAS (which restricts its search space to a set of suitable gene families). We discarded all contigs with annotations belonging to the Metazoan kingdom, to further remove host-genome sequences from further downstream analysis. We used nucleotide counts from assembled contigs with genus level taxonomic assignments to calculate Shannon entropy as a measure of diversity. We used Mann-Whitney *U* test for significance testing and subsequently adjusted the *p* values using the Bonferroni correction for multiple testing. To assess SNPs in the extracted pathogens, the FreeBayes tool was used (version 1.1.0, http://arxiv.org/abs/1207.3907). Fastq read pairs used for contig assembly were mapped back to contigs using bwa, version bwakit-0.7.12, under standard paired-end setting. Next, FreeBayes was used to call variants. Ploidy was set to 10 to predict variants present at differing frequencies, and the minimum read count for supporting alternate alleles was set to 3. The predicted variants were further filtered with the help of VcfFilter (available with the FreeBayes package), using a minimum quality score of 30 and minimum total read depth of 6 reads per position.

### Contig binning via “entropy landscapes”

We recruited all paired-end reads that had contributed to the assembly process back against the non-human contigs using Bowtie2. This recruitment was used to calculate the average number of mismatches and gaps over the length of the contig, per base pair (entropy). This score was depicted on the z-axis of the plots, together with contig length on the x-axis, and GC content on the y-axis. The axes “entropy” and “GC content” are intrinsically normalized, i.e., largely independent of the number of reads per sample (or per contig). In contrast, the axis “contig size” is not normalized. Contigs from genera constituting more than 5% of the annotated non-human contigs were color-coded according to their annotation. We depicted the unannotated and the remaining contigs from the low abundance genera in a single color. The code for this analysis is available at https://github.com/rfeigelman/Microbe-Entropy-Analysis.git.

### MLST and phylogenetic placement of abundant clonal species

We constructed a maximum likelihood tree for *S. maltophilia* using RAxML [75] under the GTRCAT model, with 1000 bootstraps, using the concatenated sequence composed of the seven housekeeping genes *atpD*, *gapA*, *guaA*, *mutM*, *nuoD*, *ppsA*, and *recA*. In patient CF-00, these genes showed 100% identity over their entire length to a previously observed strain. All the sequences of the previously typed strains used for building the tree were downloaded from http://pubmlst.org/smaltophilia/. The tree was rooted using *Xanthomonas Campestris* 8004 [GenBank accession NC_007086.1] as an outgroup. For the *Achromobacter* genus, we built another phylogenetic tree using the concatenated sequences of the seven housekeeping genes *eno*, *gltB*, *lepA*, *nrdA*, *nuoL*, *nusA*, and *rpoB*. Full-length sequences of all the seven genes were recovered from the assembled contigs of the patient CF-85. There was no exact match found to any previously documented strain. The sequences of previously typed strains used for building the tree were downloaded from http://pubmlst.org/achromobacter/. The tree was rooted using *Bordetella pertussis* [GenBank accession LN849008] as an outgroup.

### Identification of antibiotic resistance genes and cassettes

We used the Comprehensive Antibiotic Resistance Database [76] to search for resistance-conferring genes in our samples. The results from the web server were filtered to include only matches with over 90 amino acid length and over 90% identity. We further compared these findings with the clinical laboratory reports on observed antibiotic resistances.

### Genome alignment

We used Mauve [77] for ordering the assembled *Stenotrophomonas* contigs from the patient sample CF-00 and subsequently performing whole-genome alignments against reference strains.

## Additional files

Additional file 1: Table S4. Table showing the CF and COPD patient demographics data. (XLSX 41 kb)
Additional file 2:
**Figure S1.** Plot showing the sequencing throughput for each sample. Subject ID on X-axis and total number of read pairs on Y-axis. CF-99 was sampled twice. **Figure S2.** Plot showing the percentage of non-human DNA per milliliter vs. total DNA concentration. CF samples are marked in red, COPD in blue, healthy in green, and smokers in light blue. **Figure S3.** Plot showing the Shannon entropy of each sample from the four subject groups. **Figure S4.** Genus level taxonomic composition of each sample. All genera constituting less than 4.5% of the annotated fraction have been labeled as others. **Figure S5.** CF, COPD, healthy, smoker—entropy landscape showing lung microbial composition for the samples from different subject groups. **Figure S6.** Phylogenetic tree showing placement of reference *S. maltophilia* database strains in addition to the strain isolated from our subject CF-00 and all strains documented in the PubMLST database. **Figure S7.** Taxonomic summary, per sample, of total sequencing reads before assembly. Only a relatively small fraction of reads (between 2 and 5%) can be reliably assigned a non-human taxonomy. This number is somewhat higher after assembly (not shown here). (PDF 2763 kb)
Additional file 3: Table S1. Table showing assembly statistics for each sample. All reported statistics are based on contigs of size >=500 bp unless otherwise stated in the header, e.g., # contigs >=0 bp include all the assembled contigs in the statistic. Table S5 Table showing clinically tested antibiotics for detecting resistance and results from genomic analysis for available CF patients. (PDF 308 kb)
Additional file 4: Table S2. Table showing clinically detected bacteria and their subsequent identification using genomic analysis. (XLSX 42 kb)
Additional file 5: Table S3. Table showing number of assembled nucleotides from each subject sample and their corresponding taxonomy. (XLS 114 kb)
Additional file 6: Table S7 Table summarizing strain-level variation for the 11 best-covered pathogens, subdivided per patient. (XLSX 40 kb)
Additional file 7: Table S6. Table summarizing and comparing the taxonomy assignments by homology searches against the NCBI nucleotide database on the one hand and by the MiDAS analysis tool on the other hand. (XLSX 220 kb)

## Abbreviations

CF: Cystic fibrosis
CFTR: Cystic fibrosis transmembrane regulator
COPD: Chronic obstructive pulmonary disease
DNA: Deoxyribonucleic acid
GC: Guanine-cytosine
MDR: Multidrug-resistant
MLST: Multi-locus sequence typing
PCR: Polymerase chain reaction
RNA: Ribonucleic acid
WGS: Whole-genome shotgun sequencing

## References

[1] Walters S, Mehta A. Epidemiology of cystic fibrosis. In: Hodson M, Geddes DM, Bush A, editors. Cystic fibrosis, 3rd edn. London: Edward Arnold Ltd; 2007. p. 21–45.

[2] Boucher RC (2002). An overview of the pathogenesis of cystic fibrosis lung disease. Adv Drug Deliv Rev.

[3] Mahenthiralingam E (2014). Emerging cystic fibrosis pathogens and the microbiome. Paediatr Respir Rev.

[4] Bell SC, De Boeck K, Amaral MD (2015). New pharmacological approaches for cystic fibrosis: promises, progress, pitfalls. Pharmacol Ther.

[5] Laura GAO, Filkins M (2015). Cystic fibrosis lung infections: polymicrobial, complex, and hard to treat.

[6] Tan K, Conway SP, Brownlee KG, Etherington C, Peckham DG (2002). Alcaligenes infection in cystic fibrosis. Pediatr Pulmonol.

[7] Talmaciu I, Varlotta L, Mortensen J, Schidlow DV (2000). Risk factors for emergence of Stenotrophomonas maltophilia in cystic fibrosis. Pediatr Pulmonol.

[8] Zhou J, Garber E, Desai M, Saiman L (2006). Compliance of clinical microbiology laboratories in the United States with current recommendations for processing respiratory tract specimens from patients with cystic fibrosis. J Clin Microbiol.

[9] Surette MG (2014). The cystic fibrosis lung microbiome. Ann Am Thorac Soc.

[10] Chmiel JF, Aksamit TR, Chotirmall SH, Dasenbrook EC, Elborn JS, LiPuma JJ, Ranganathan SC, Waters VJ, Ratjen FA (2014). Antibiotic management of lung infections in cystic fibrosis. I. The microbiome, methicillin-resistant Staphylococcus aureus, gram-negative bacteria, and multiple infections. Ann Am Thorac Soc.

[11] Dickson RP, Erb-Downward JR, Martinez FJ, Huffnagle GB (2016). The microbiome and the respiratory tract. Annu Rev Physiol.

[12] Coburn B, Wang PW, Diaz Caballero J, Clark ST, Brahma V, Donaldson S, Zhang Y, Surendra A, Gong Y, Elizabeth Tullis D, Yau YCW, Waters VJ, Hwang DM, Guttman DS (2015). Lung microbiota across age and disease stage in cystic fibrosis. Sci Rep.

[13] Whiteson KL, Bailey B, Bergkessel M, Conrad D, Delhaes L, Felts B, Harris JK, Hunter R, Lim YW, Maughan H, Quinn R, Salamon P, Sullivan J, Wagner BD, Rainey PB (2014). The upper respiratory tract as a microbial source for pulmonary infections in cystic fibrosis. Parallels from island biogeography. Am J Respir Crit Care Med.

[14] Moran Losada P, Chouvarine P, Dorda M, Hedtfeld S, Mielke S, Schulz A, Wiehlmann L, Tummler B (2016). The cystic fibrosis lower airways microbial metagenome. ERJ Open Res.

[15] Quinn RA, Lim YW, Maughan H, Conrad D, Rohwer F, Whiteson KL (2014). Biogeochemical forces shape the composition and physiology of polymicrobial communities in the cystic fibrosis lung. MBio.

[16] Lim YW, Haynes M, Furlan M, Robertson CE, Harris JK, Rohwer F (2014). Purifying the impure: sequencing metagenomes and metatranscriptomes from complex animal-associated samples. J Vis Exp..

[17] Lim YW, Schmieder R, Haynes M, Willner D, Furlan M, Youle M, Abbott K, Edwards R, Evangelista J, Conrad D, Rohwer F (2013). Metagenomics and metatranscriptomics: windows on CF-associated viral and microbial communities. J Cyst Fibros.

[18] Lim YW, Evangelista JS, Schmieder R, Bailey B, Haynes M, Furlan M, Maughan H, Edwards R, Rohwer F, Conrad D, Forbes BA (2014). Clinical insights from metagenomic analysis of sputum samples from patients with cystic fibrosis. J Clin Microbiol.

[19] Smith AL, Redding G, Doershuk C, Goldmann D, Gore E, Hilman B, Marks M, Moss R, Ramsey B, Roblo T, Schwartz RH, Thomassen MJ, Williams-Warren J, Weber A, Wilmott RW, Wilson HD, Yogev R (1988). Sputum changes associated with therapy for endobronchial exacerbation in cystic fibrosis. J Pediatr.

[20] Dhooghe B, Noël S, Huaux F, Leal T (2014). Lung inflammation in cystic fibrosis: pathogenesis and novel therapies. Clin Biochem.

[21] Charlson ES, Bittinger K, Chen J, Diamond JM, Li H, Collman RG, Bushman FD (2012). Assessing bacterial populations in the lung by replicate analysis of samples from the upper and lower respiratory tracts. PLoS ONE.

[22] Charlson ES, Bittinger K, Haas AR, Fitzgerald AS, Frank I, Yadav A, Bushman FD, Collman RG (2011). Topographical continuity of bacterial populations in the healthy human respiratory tract. Am J Respir Crit Care Med.

[23] Bassis CM, Erb-Downward JR, Dickson RP, Freeman CM, Schmidt TM, Young VB, Beck JM, Curtis JL, Huffnagle GB (2015). Analysis of the upper respiratory tract microbiotas as the source of the lung and gastric microbiotas in healthy individuals. MBio.

[24] Venkataraman A, Bassis CM, Beck JM, Young VB, Curtis JL, Huffnagle GB, Schmidt TM (2015). Application of a neutral community model to assess structuring of the human lung microbiome. MBio.

[25] Costello A, Reen FJ, O'Gara F, Callaghan M, McClean S (2014). Inhibition of co-colonizing cystic fibrosis-associated pathogens by Pseudomonas aeruginosa and Burkholderia multivorans. Microbiology (Reading, Engl).

[26] Zhao J, Schloss PD, Kalikin LM, Carmody LA, Foster BK, Petrosino JF, Cavalcoli JD, VanDevanter DR, Murray S, Li JZ, Young VB, LiPuma JJ (2012). Decade-long bacterial community dynamics in cystic fibrosis airways. Proc Natl Acad Sci U S A.

[27] Carmody LA, Zhao J, Schloss PD, Petrosino JF, Murray S, Young VB, Li JZ, LiPuma JJ (2013). Changes in cystic fibrosis airway microbiota at pulmonary exacerbation. Ann Am Thorac Soc.

[28] Brown PS, Pope CE, Marsh RL, Qin X, McNamara S, Gibson R, Burns JL, Deutsch G, Hoffman LR (2014). Directly sampling the lung of a young child with cystic fibrosis reveals diverse microbiota. Ann Am Thorac Soc.

[29] Fodor AA, Klem ER, Gilpin DF, Elborn JS, Boucher RC, Tunney MM, Wolfgang MC (2012). The adult cystic fibrosis airway microbiota is stable over time and infection type, and highly resilient to antibiotic treatment of exacerbations. PLoS ONE.

[30] Tunney MM, Klem ER, Fodor AA, Gilpin DF, Moriarty TF, McGrath SJ, Muhlebach MS, Boucher RC, Cardwell C, Doering G, Elborn JS, Wolfgang MC (2011). Use of culture and molecular analysis to determine the effect of antibiotic treatment on microbial community diversity and abundance during exacerbation in patients with cystic fibrosis. Thorax.

[31] Cramer N, Wiehlmann L, Tümmler B (2010). Clonal epidemiology of Pseudomonas aeruginosa in cystic fibrosis. Int J Med Microbiol.

[32] Coutinho CP, Dos Santos SC, Madeira A, Mira NP, Moreira AS, Sá-Correia I (2011). Long-term colonization of the cystic fibrosis lung by Burkholderia cepacia complex bacteria: epidemiology, clonal variation, and genome-wide expression alterations. Front Cell Infect Microbiol.

[33] Maiden MCJ, van Rensburg MJ, Bray JE, Earle SG, Ford SA, Jolley KA, McCarthy ND (2013). MLST revisited: the gene-by-gene approach to bacterial genomics. Nat Rev Microbiol.

[34] Brooke JS (2012). Stenotrophomonas maltophilia: an emerging global opportunistic pathogen. Clin Microbiol Rev.

[35] Martínez JL (2008). Antibiotics and antibiotic resistance genes in natural environments. Science.

[36] Gherardi G, Creti R, Pompilio A, Di Bonaventura G (2015). Diagnostic microbiology and infectious disease. Diagn Microbiol Infect Dis.

[37] Parks DH, Imelfort M, Skennerton CT, Hugenholtz P, Tyson GW (2015). CheckM: assessing the quality of microbial genomes recovered from isolates, single cells, and metagenomes. Genome Res.

[38] Hansen CR, Pressler T, Nielsen KG, Jensen PO, Bjarnsholt T, Hoiby N (2010). Inflammation in Achromobacter xylosoxidans infected cystic fibrosis patients. J Cyst Fibros.

[39] Amoureux L, Bador J, Fardeheb S, Mabille C, Couchot C, Massip C, Salignon AL, Berlie G, Varin V, Neuwirth C (2013). Detection of Achromobacter xylosoxidans in hospital, domestic, and outdoor environmental samples and comparison with human clinical isolates. Appl Environ Microbiol.

[40] De Baets F, Schelstraete P, Van Daele S, Haerynck F, Vaneechoutte M (2007). Achromobacter xylosoxidans in cystic fibrosis: prevalence and clinical relevance. J Cyst Fibros.

[41] Rønne Hansen C, Pressler T, Høiby N, Gormsen M (2006). Chronic infection with Achromobacter xylosoxidans in cystic fibrosis patients; a retrospective case control study. J Cyst Fibros.

[42] Spilker T, Vandamme P, LiPuma JJ (2013). Identification and distribution of Achromobacter species in cystic fibrosis. J Cyst Fibros.

[43] Jolley KA, Maiden MCJ (2010). BIGSdb: scalable analysis of bacterial genome variation at the population level. BMC Bioinformatics.

[44] Ridderberg W, Wang M, Norskov-Lauritsen N (2012). Multilocus sequence analysis of isolates of Achromobacter from patients with cystic fibrosis reveals infecting species other than Achromobacter xylosoxidans. J Clin Microbiol.

[45] Cystic Fibrosis Foundation (2015). Cystic fibrosis patient registry 2014 annual data report.

[46] Yang L, Jelsbak L, Marvig RL, Damkiær S, Workman CT, Rau MH, Hansen SK, Folkesson A, Johansen HK, Ciofu O, Høiby N, Sommer MOA, Molin S (2011). Evolutionary dynamics of bacteria in a human host environment.

[47] Peschel A, Jack RW, Otto M, Collins LV, Staubitz P, Nicholson G, Kalbacher H, Nieuwenhuizen WF, Jung G, Tarkowski A, van Kessel KP, van Strijp JA (2001). Staphylococcus aureus resistance to human defensins and evasion of neutrophil killing via the novel virulence factor MprF is based on modification of membrane lipids with l-lysine. J Exp Med.

[48] Lieberman TD, Flett KB, Yelin I, Martin TR, McAdam AJ, Priebe GP, Kishony R (2014). Genetic variation of a bacterial pathogen within individuals with cystic fibrosis provides a record of selective pressures. Nat Genet.

[49] Diaz Caballero J, Clark ST, Coburn B, Zhang Y, Wang PW, Donaldson SL, Tullis DE, Yau YCW, Waters VJ, Hwang DM, Guttman DS (2015). Selective sweeps and parallel pathoadaptation drive pseudomonas aeruginosa evolution in the cystic fibrosis lung. MBio.

[50] Marvig RL, Sommer LM, Molin S, Johansen HK (2014). Convergent evolution and adaptation of Pseudomonas aeruginosa within patients with cystic fibrosis. Nat Genet.

[51] Pulcrano G, Iula DV, Raia V, Rossano F, Catania MR (2012). Different mutations in mucA gene of Pseudomonas aeruginosa mucoid strains in cystic fibrosis patients and their effect on algU gene expression. New Microbiol.

[52] Li Z, Kosorok MR, Farrell PM, Laxova A, West SEH, Green CG, Collins J, Rock MJ, Splaingard ML (2005). Longitudinal development of mucoid Pseudomonas aeruginosa infection and lung disease progression in children with cystic fibrosis. JAMA.

[53] Pukatzki S, Ma AT, Revel AT, Sturtevant D, Mekalanos JJ (2007). Type VI secretion system translocates a phage tail spike-like protein into target cells where it cross-links actin. Proc Natl Acad Sci U S A.

[54] Hood RD, Singh P, Hsu F, Güvener T, Carl MA, Trinidad RRS, Silverman JM, Ohlson BB, Hicks KG, Plemel RL, Li M, Schwarz S, Wang WY, Merz AJ, Goodlett DR, Mougous JD (2010). A type VI secretion system of Pseudomonas aeruginosa targets a toxin to bacteria. Cell Host and Microbe.

[55] Bingle LE, Bailey CM, Pallen MJ (2008). Type VI secretion: a beginner’s guide. Curr Opin Microbiol.

[56] Boyer F, Fichant G, Berthod J, Vandenbrouck Y, Attree I (2009). Dissecting the bacterial type VI secretion system by a genome wide in silico analysis: what can be learned from available microbial genomic resources?. BMC Genomics.

[57] Ryan RP, Monchy S, Cardinale M, Taghavi S, Crossman L, Avison MB, Berg G, van der Lelie D, Dow JM (2009). The versatility and adaptation of bacteria from the genus Stenotrophomonas. Nat Rev Microbiol.

[58] Ormerod KL, George NM, Fraser JA, Wainwright C, Hugenholtz P (2015). Comparative genomics of non-pseudomonal bacterial species colonising paediatric cystic fibrosis patients. Peer J.

[59] Alavi P, Starcher MR, Thallinger GG, Zachow C, ller HM, Berg G. Stenotrophomonas comparative genomics reveals genes and functions that differentiate beneficial and pathogenic bacteria. BMC Genomics. 2014;15(1):482. 10.1186/1471-2164-15-482 PMC4101175 24939220

[60] Adamek M, Linke B, Schwartz T (2014). Virulence genes in clinical and environmental Stenotrophomas maltophilia isolates: a genome sequencing and gene expression approach. Microb Pathog.

[61] Baron C (2010). Antivirulence drugs to target bacterial secretion systems. Curr Opin Microbiol.

[62] Rogers GB, Stressmann FA, Koller G, Daniels T, Carroll MP, Bruce KD (2008). Assessing the diagnostic importance of nonviable bacterial cells in respiratory infections. Diagn Microbiol Infect Dis.

[63] Nelson KE, Weinstock GM, Highlander SK, Worley KC, Creasy HH, Wortman JR, Rusch DB, Mitreva M, Sodergren E, Chinwalla AT, Feldgarden M, Gevers D, Haas BJ, Madupu R, Ward DV, Birren BW, Gibbs RA, Methé B, Petrosino JF, Strausberg RL, Sutton GG, White OR, Wilson RK, Durkin S, Giglio MG, Gujja S, Howarth C, Kodira CD, Kyrpides N, Mehta T, Muzny DM, Pearson M, Pepin K, Pati A, Qin X, Yandava C, Zeng Q, Zhang L, Berlin AM, Chen L, Hepburn TA, Johnson J, McCorrison J, Miller J, Minx P, Nusbaum C, Russ C, Sykes SM, Tomlinson CM, Young S, Warren WC, Badger J, Crabtree J, Markowitz VM, Orvis J, Cree A, Ferriera S, Fulton LL, Fulton RS, Gillis M, Hemphill LD, Joshi V, Kovar C, Torralba M, Wetterstrand KA, Abouellleil A, Wollam AM, Buhay CJ, Ding Y, Dugan S, FitzGerald MG, Holder M, Hostetler J, Clifton SW, Allen-Vercoe E, Earl AM, Farmer CN, Liolios K, Surette MG, Xu Q, Pohl C, Wilczek-Boney K, Zhu D, Human Microbiome Jumpstart Reference Strains Consortium (2010). A catalog of reference genomes from the human microbiome. Science.

[64] Wylie KM, Truty RM, Sharpton TJ, Mihindukulasuriya KA, Zhou Y, Gao H, Sodergren E, Weinstock GM, Pollard KS (2012). Novel bacterial taxa in the human microbiome. PLoS ONE.

[65] Chatterji S, Yamazaki I, Bai Z, Eisen JA. CompostBin: a DNA composition-based algorithm for binning environmental shotgun reads. Research in Computational Molecular Biology. 2008:17–28. doi:10.1007/978-3-540-78839-3_3.

[66] Aaron SD, Vandemheen KL, Ferris W, Fergusson D, Tullis E, Haase D, Berthiaume Y, Brown N, Wilcox P, Yozghatlian V, Bye P, Bell S, Chan F, Rose B, Jeanneret A, Stephenson A, Noseworthy M, Freitag A, Paterson N, Doucette S, Harbour C, Ruel M, MacDonald N (2005). Combination antibiotic susceptibility testing to treat exacerbations of cystic fibrosis associated with multiresistant bacteria: a randomised, double-blind, controlled clinical trial. Lancet.

[67] Keays T, Ferris W, Vandemheen KL, Chan F, Yeung S-W, Mah T-F, Ramotar K, Saginur R, Aaron SD (2009). A retrospective analysis of biofilm antibiotic susceptibility testing: a better predictor of clinical response in cystic fibrosis exacerbations. J Cyst Fibros.

[68] Martinez JL, Baquero F, Andersson DI (2007). Predicting antibiotic resistance. Nat Rev Microbiol.

[69] Bioinformatics B (2011). FastQC: a quality control tool for high throughput sequence data.

[70] Langmead B, Salzberg SL (2012). Fast gapped-read alignment with Bowtie 2. Nat Publ Group.

[71] Meyer LR, Zweig AS, Hinrichs AS, Karolchik D, Kuhn RM, Wong M, Sloan CA, Rosenbloom KR, Roe G, Rhead B (2013). The UCSC Genome Browser database: extensions and updates 2013. Nucleic Acids Res.

[72] Bankevich A, Nurk S, Antipov D, Gurevich AA, Dvorkin M, Kulikov AS, Lesin VM, Nikolenko SI, Pham S, Prjibelski AD, Pyshkin AV, Sirotkin AV, Vyahhi N, Tesler G, Alekseyev MA, Pevzner PA (2012). SPAdes: a new genome assembly algorithm and its applications to single-cell sequencing. J Comput Biol.

[73] Altschul SF, Gish W, Miller W, Myers EW, Lipman DJ (1990). Basic local alignment search tool. J Mol Biol.

[74] Nayfach S, Rodriguez-Mueller B, Garud N, Pollard KS (2016). An integrated metagenomics pipeline for strain profiling reveals novel patterns of bacterial transmission and biogeography. Genome Res.

[75] Stamatakis A (2006). RAxML-VI-HPC: maximum likelihood-based phylogenetic analyses with thousands of taxa and mixed models. Bioinformatics.

[76] McArthur AG, Waglechner N, Nizam F, Yan A, Azad MA, Baylay AJ, Bhullar K, Canova MJ, De Pascale G, Ejim L, Kalan L, King AM, Koteva K, Morar M, Mulvey MR, O'Brien JS, Pawlowski AC, Piddock LJV, Spanogiannopoulos P, Sutherland AD, Tang I, Taylor PL, Thaker M, Wang W, Yan M, Yu T, Wright GD (2013). The comprehensive antibiotic resistance database. Antimicrob Agents Chemother.

[77] Darling ACE, Mau B, Blattner FR, Perna NT (2004). Mauve: multiple alignment of conserved genomic sequence with rearrangements. Genome Res.

