# Supplementary material for: Sputum DNA sequencing in cystic fibrosis: non-invasive access to the lung microbiome and to pathogen details
Source: Microbiome. 2017 Feb 10;5:20. doi: 10.1186/s40168-017-0234-1 (PMC5303297; doi:10.1186/s40168-017-0234-1)
Supplement: Additional file 2: — Figure S1. Plot showing the sequencing throughput for each sample. Subject ID on X-axis and total number of read pairs on Y-axis. CF-99 was sampled twice. Figure S2. Plot showing the percentage of non-human DNA per milliliter vs. total DNA concentration. CF samples are marked in red, COPD in blue, healthy in green, and smokers in light blue. Figure S3. Plot showing the Shannon entropy of each sample from the four subject groups. Figure S4. Genus level taxonomic composition of each sample. All genera constituting less than 4.5% of the annotated fraction have been labeled as others. Figure S5. CF, COPD, healthy, smoker—entropy landscape showing lung microbial composition for the samples from different subject groups. Figure S6. Phylogenetic tree showing placement of reference S. maltophilia database strains in addition to the strain isolated from our subject CF-00 and all strains documented in the PubMLST database. Figure S7. Taxonomic summary, per sample, of total sequencing reads before assembly. Only a relatively small fraction of reads (between 2 and 5%) can be reliably assigned a non-human taxonomy. This number is somewhat higher after assembly (not shown here). (PDF 2763 kb) [file 40168_2017_234_MOESM2_ESM.pdf]

Figure S1

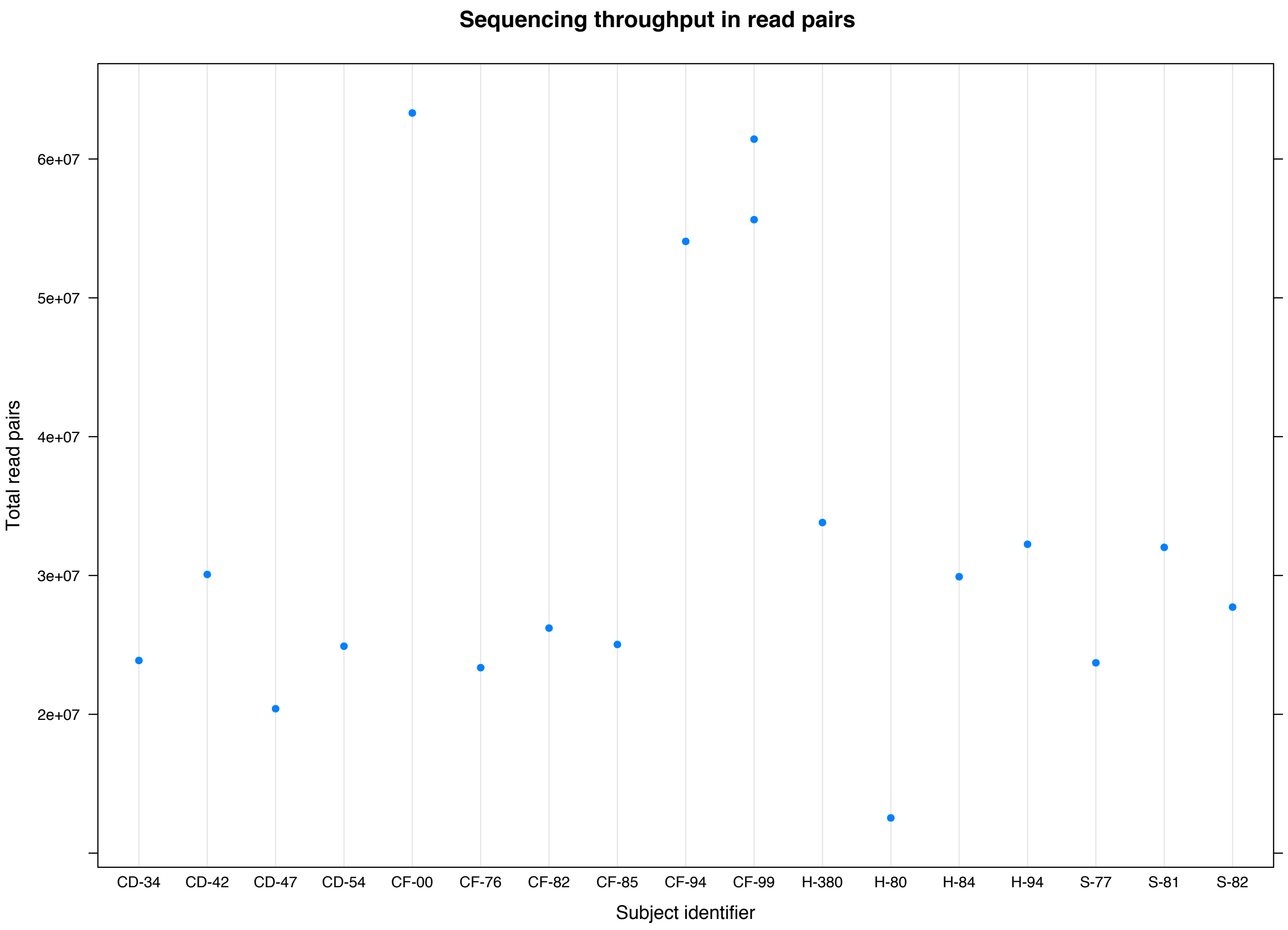

Sequencing throughput in read pairs
Total read pairs
6e+07
5e+07
4e+07
3e+07
2e+07
CD-34
CD-42
CD-47
CD-54
CF-00
CF-76
CF-82
CF-85
CF-94
CF-99
H-380
H-80
H-84
H-94
S-77
S-81
S-82
Subject identifier

Figure S2

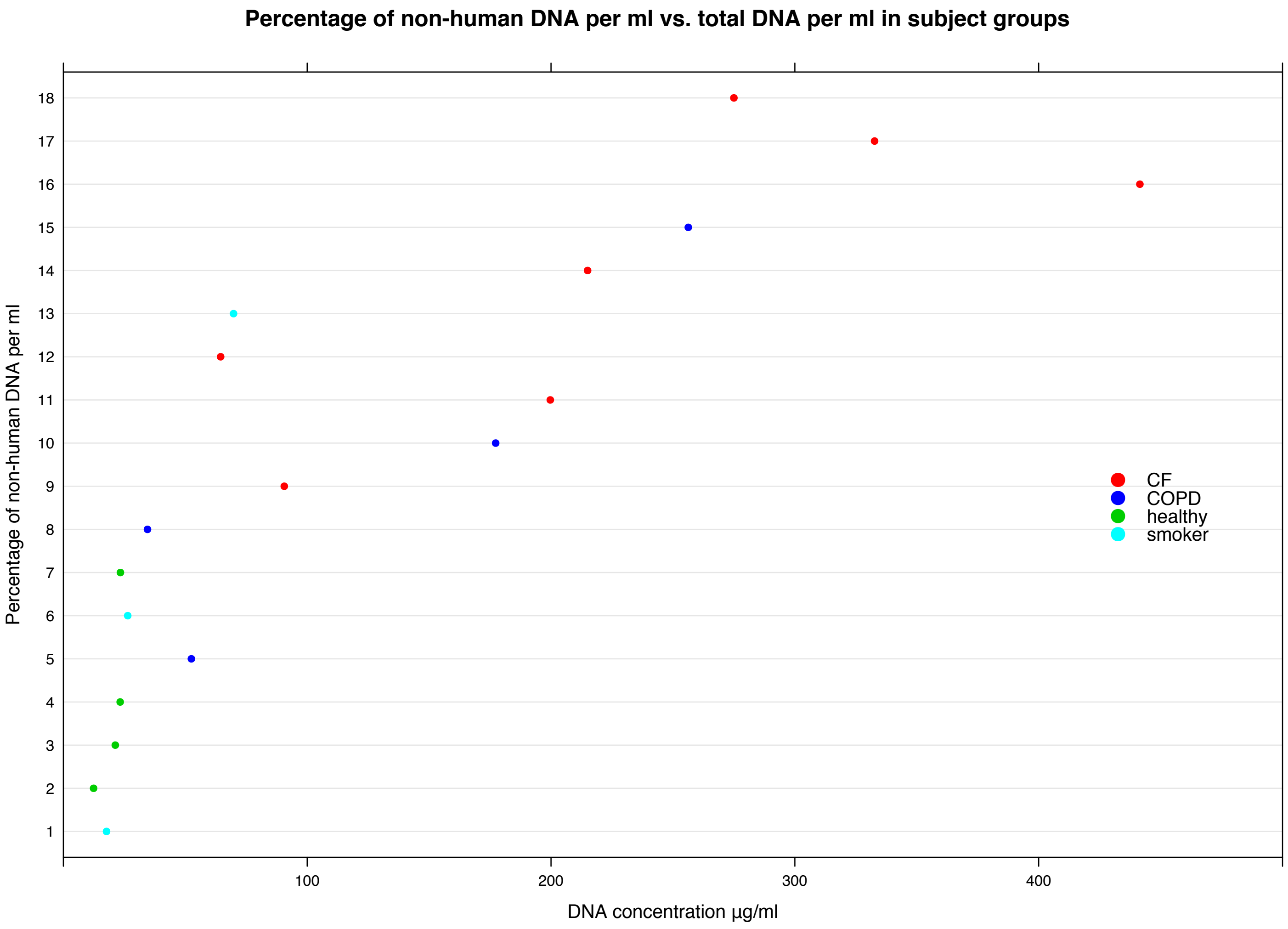
Percentage of non-human DNA per ml vs. total DNA per ml in subject groups
Percentage of non-human DNA per ml
1
2
3
4
5
6
7
8
9
10
11
12
13
14
15
16
17
18
100
200
300
400
DNA concentration µg/ml
CF
COPD
healthy
smoker

Figure S3

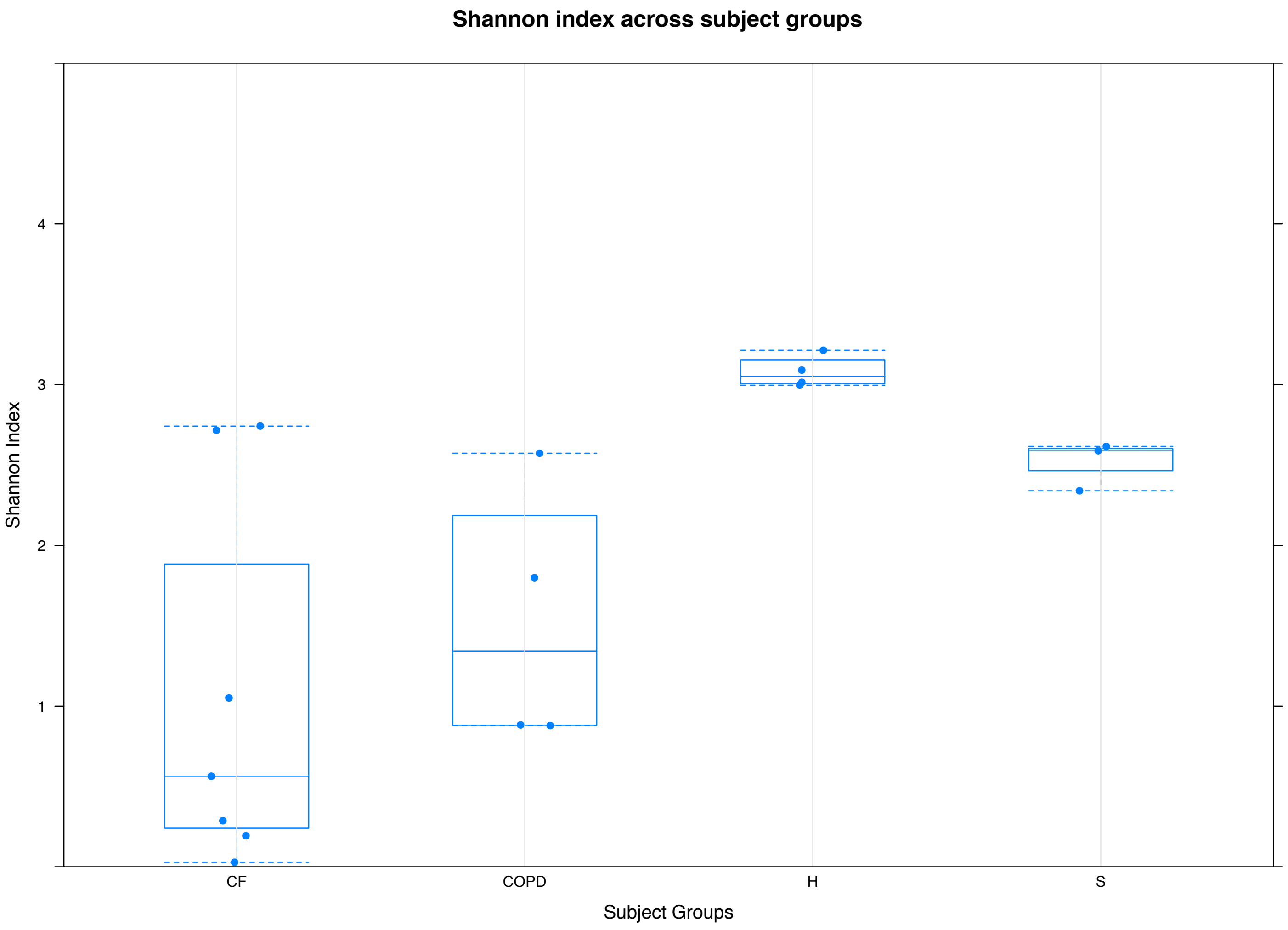
Shannon index across subject groups
Shannon Index
4
3
2
1
CF
COPD
H
S
Subject Groups

Figure S4

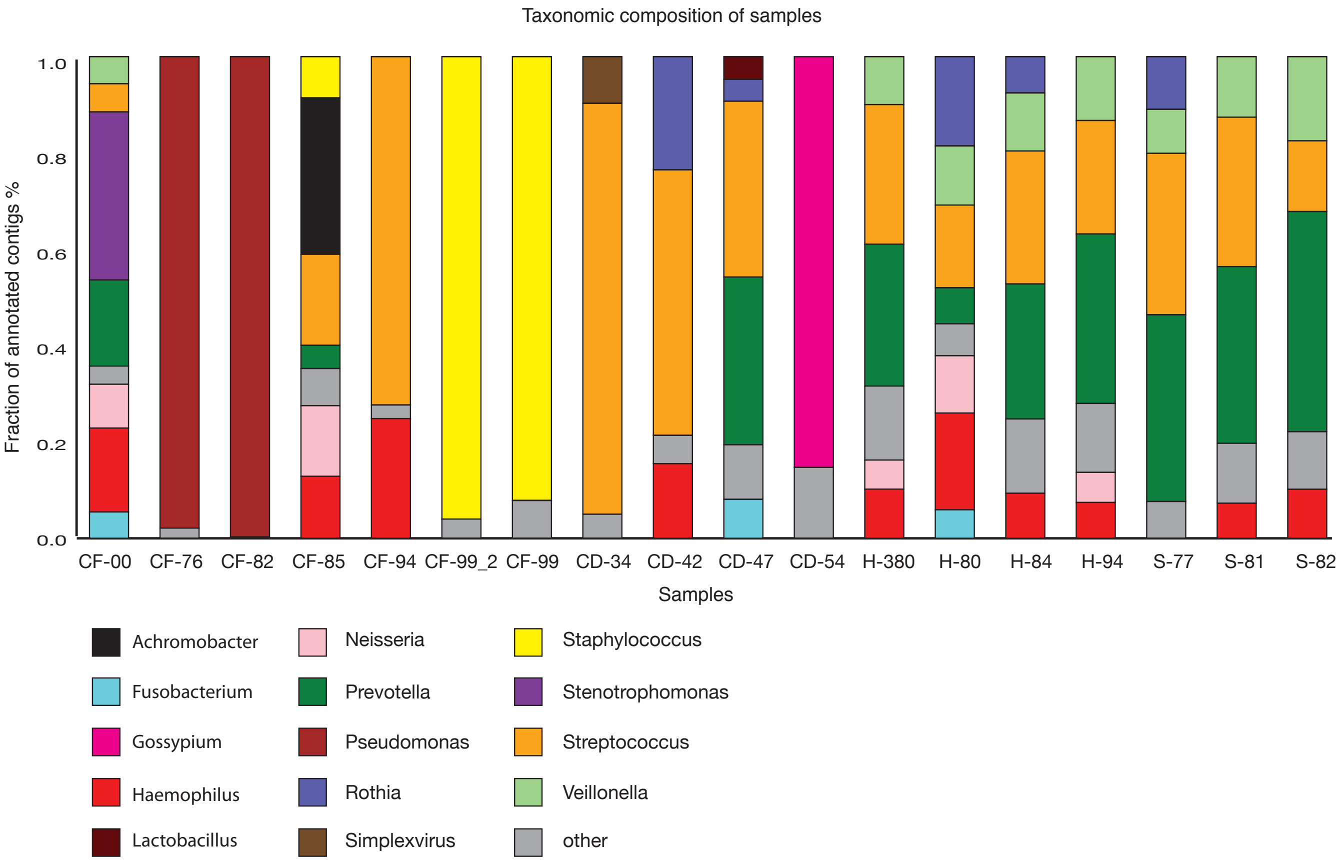

Taxonomic composition of samples
Fraction of annotated contigs %
1.0
0.8
0.6
0.4
0.2
0.0
CF-00
CF-76
CF-82
CF-85
CF-94
CF-99_2
CF-99
CD-34
CD-42
CD-47
CD-54
H-380
H-80
H-84
H-94
S-77
S-81
S-82
Samples
Achromobacter
Fusobacterium
Gossypium
Haemophilus
Lactobacillus
Neisseria
Prevotella
Pseudomonas
Rothia
Simplexvirus
Staphylococcus
Stenotrophomonas
Streptococcus
Veillonella
other

Figure S5

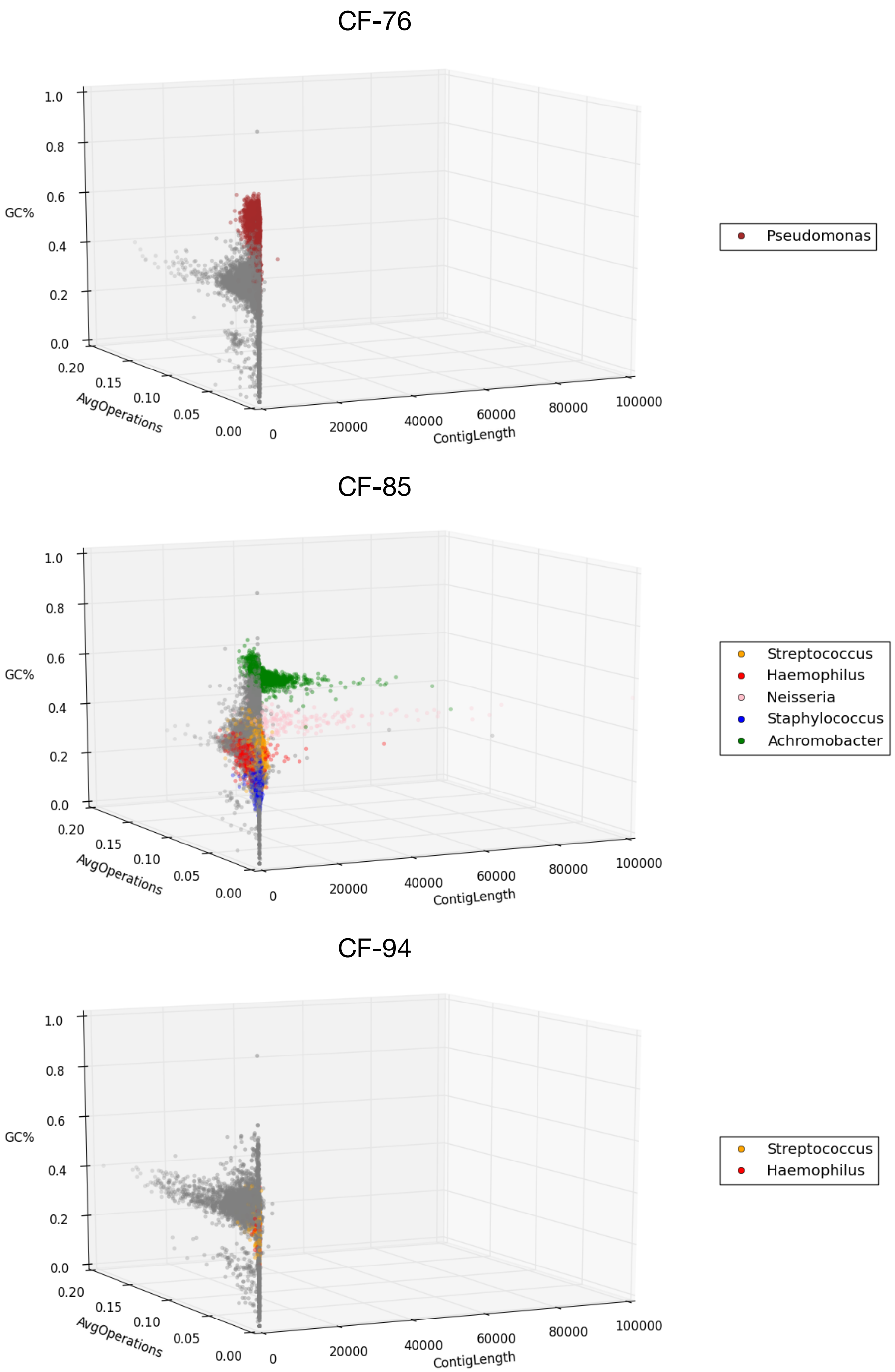
CF-76
GC%
AvgOperations
ContigLength
1.0
0.8
0.6
0.4
0.2
0.0
0.20
0.15
0.10
0.05
0.00
0
20000
40000
60000
80000
100000
Pseudomonas
CF-85
GC%
AvgOperations
ContigLength
Streptococcus
Haemophilus
Neisseria
Staphylococcus
Achromobacter
CF-94
GC%
AvgOperations
ContigLength
Streptococcus
Haemophilus

Figure S5 - continued

CF-99_2

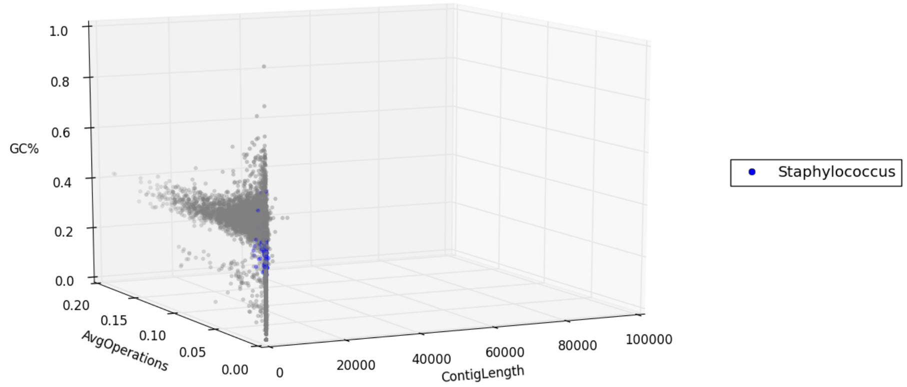

CF-99

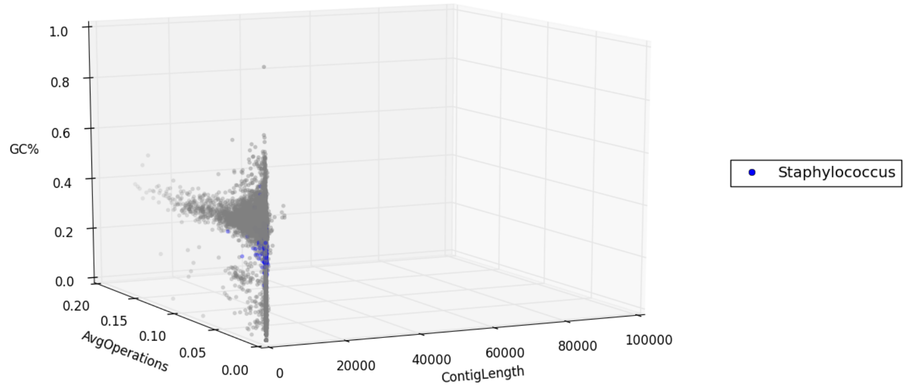

CD-34

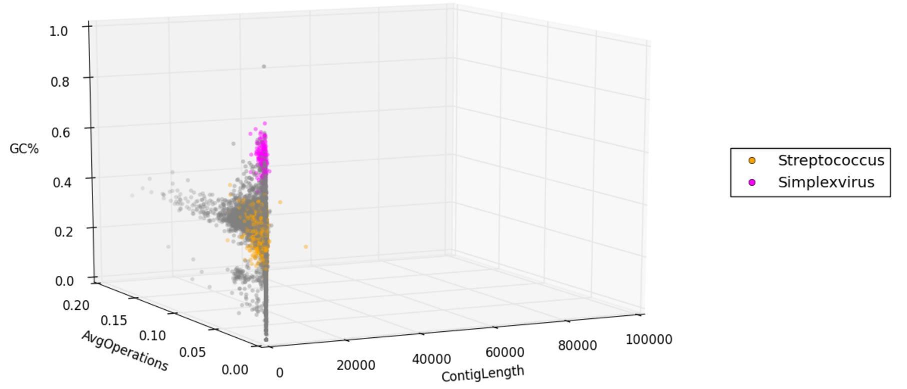

Figure S5 - continued

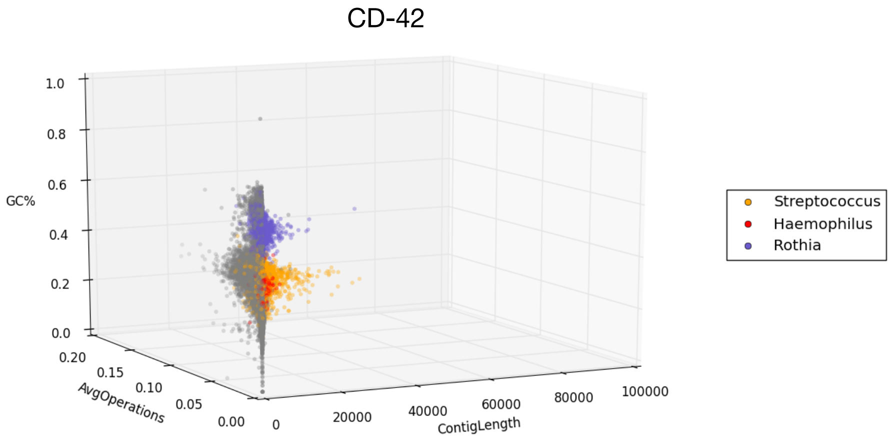

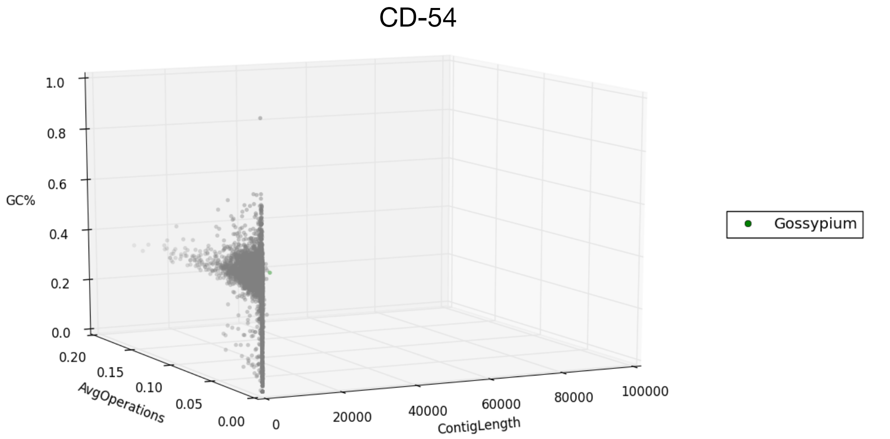

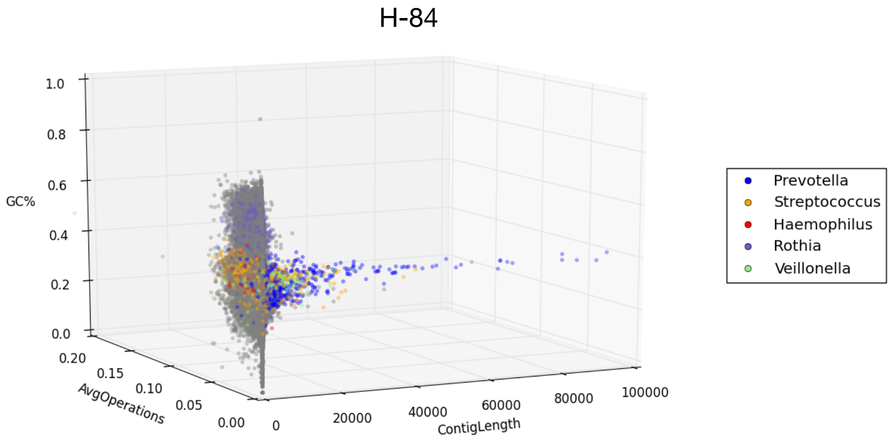

Figure S5 - continued

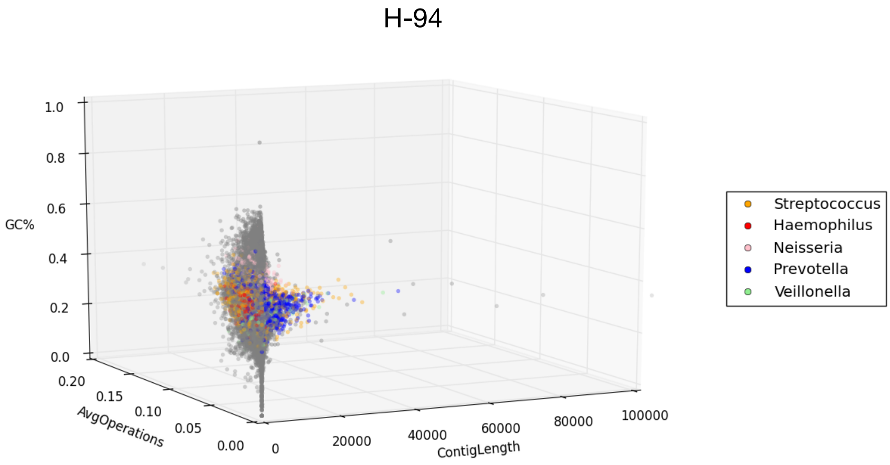

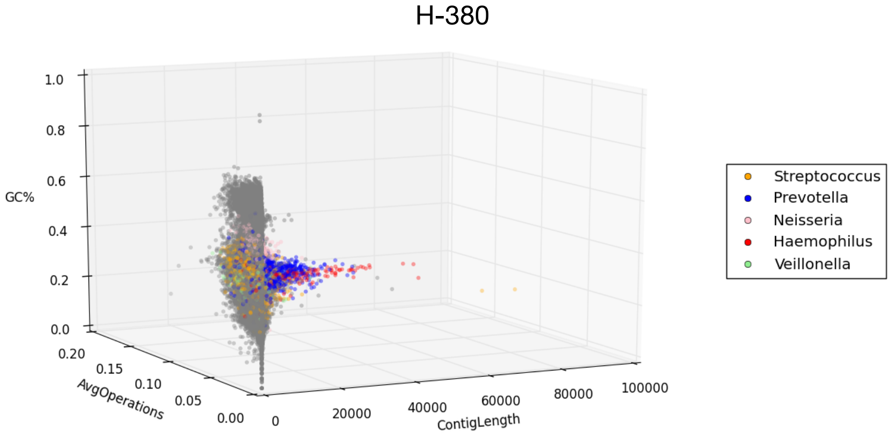

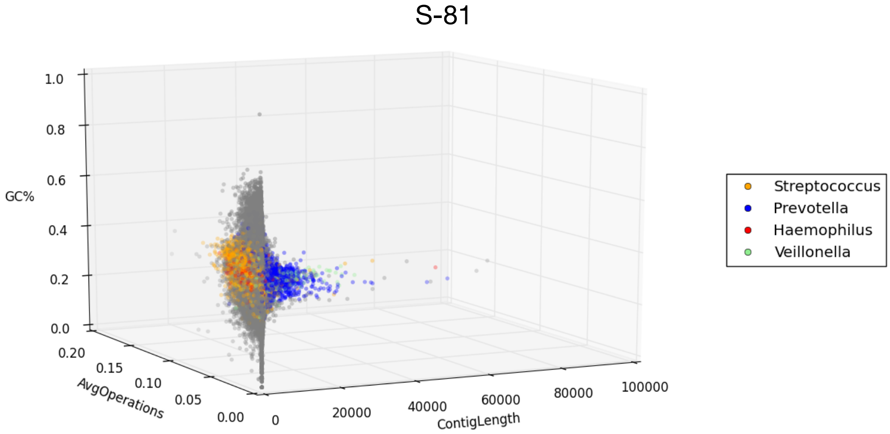

Figure S5 - continued

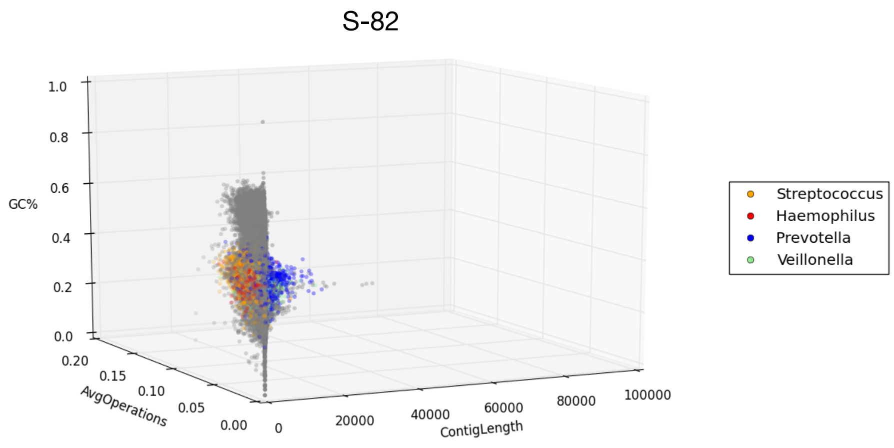

Figure S6

Phylogenetic Tree showing all the known *S. maltophila* strains and reference genomes

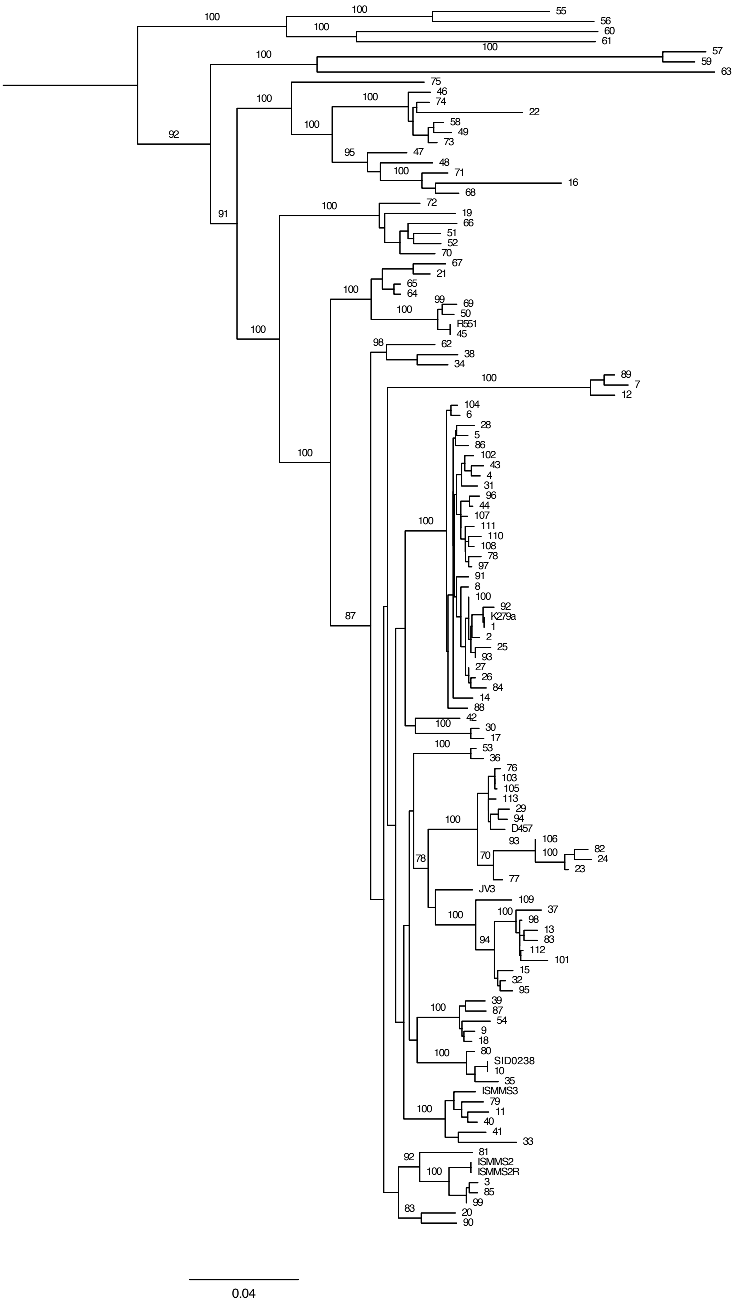

Figure S7

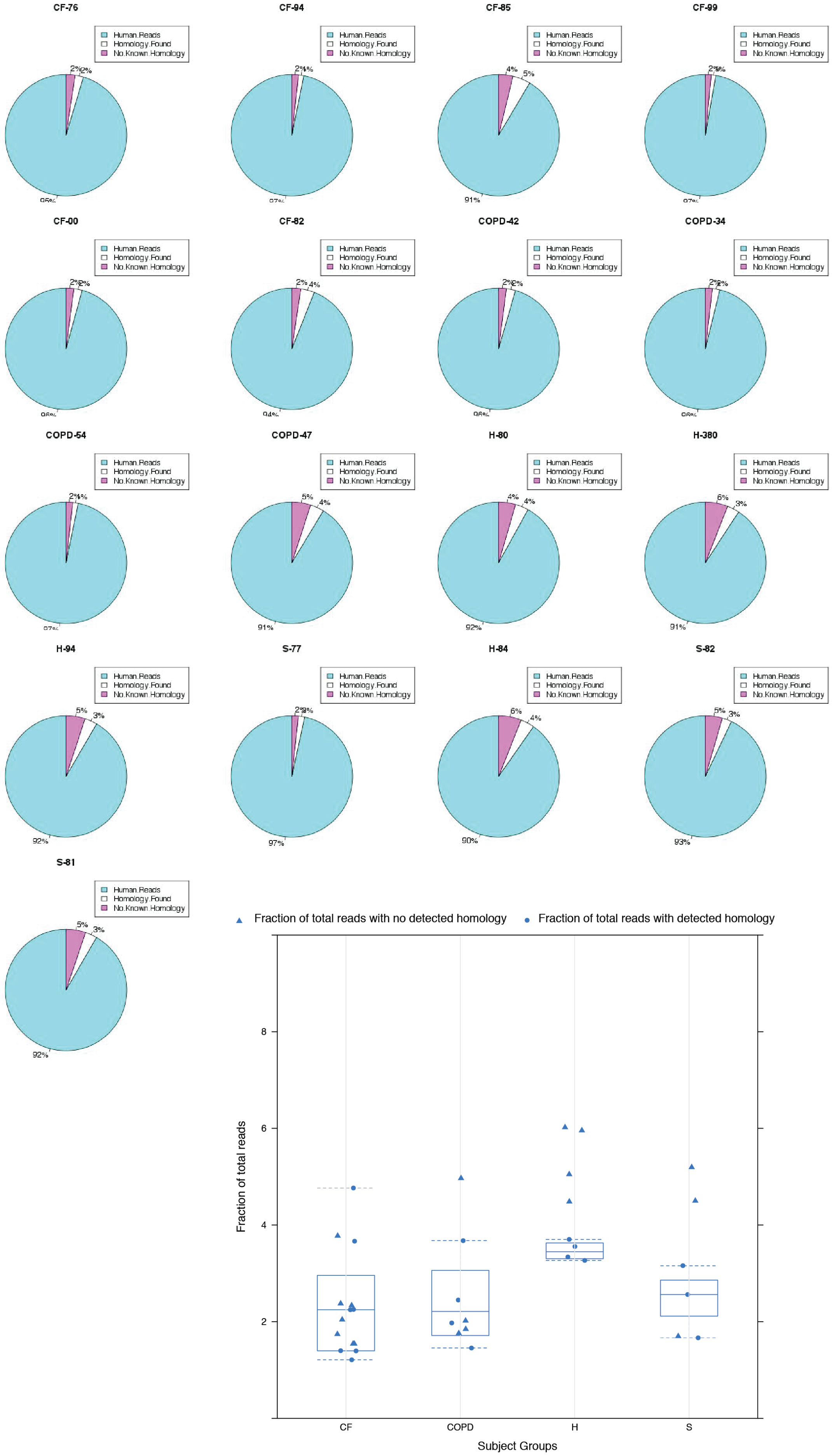
CF-76
CF-94
CF-85
CF-99
CF-00
CF-82
COPD-42
COPD-34
COPD-54
COPD-47
H-80
H-380
H-94
S-77
H-84
S-82
S-81
Human.Reads
Homology.Found
No.Known.Homology
Fraction of total reads with no detected homology
Fraction of total reads with detected homology
Fraction of total reads
2
4
6
8
CF
COPD
H
S
Subject Groups
