# Supplementary material for: Sputum DNA sequencing in cystic fibrosis: non-invasive access to the lung microbiome and to pathogen details
Source: Microbiome. 2017 Feb 10;5:20. doi: 10.1186/s40168-017-0234-1 (PMC5303297; doi:10.1186/s40168-017-0234-1)
Supplement: Additional file 3: Table S1. — Table showing assembly statistics for each sample. All reported statistics are based on contigs of size >=500 bp unless otherwise stated in the header, e.g., # contigs >=0 bp include all the assembled contigs in the statistic. Table S5 Table showing clinically tested antibiotics for detecting resistance and results from genomic analysis for available CF patients. (PDF 308 kb) [file 40168_2017_234_MOESM3_ESM.pdf]

Table S1

Assembly	# contigs (>= 0 bp)	# contigs (>= 1000 bp)	Total length (>= 0 bp)	Total length (>= 1000 bp)	# contigs	Largest contig	Total length	GC (%)	N50	N75	L50	L75	# N's per 100 kbp
CF-00	38823	3266	24786995	11805610	10749	208258	16902935	48.91	2065	874	789	4202	0
CF-76	19323	165	5671638	267724	1681	6062	1229046	47.31	675	571	607	1104	0
CF-82	60980	756	14496006	6768924	2223	125460	7735137	61.85	17599	5425	107	298	0
CF-85	35186	3198	22231999	11004261	8531	102780	14584101	52.03	2979	1017	893	3133	0
CF-94	13819	1087	5985366	1876973	3732	13906	3687264	41.43	1021	699	1055	2162	0
CF-99_2	17006	1235	6655151	2119710	3945	12761	3984350	41.74	1063	713	1112	2270	0
CF-99	16575	1130	6854419	1940694	3957	16537	3888755	41.15	998	700	1134	2309	0
COPD-34	15360	171	4605188	282938	1479	11007	1132753	41.26	716	583	513	954	0
COPD-42	33966	2873	17839126	6851544	7957	27369	10308981	43.84	1552	817	1498	3848	0
COPD-47	61667	1787	22200028	7274788	6461	570062	10329294	41.05	2985	848	473	2303	0
COPD-54	10425	188	3396474	315792	1191	7112	970067	40.59	766	600	384	746	0
H-80	35900	3506	19015599	7167982	9476	20548	11265202	48.08	1308	791	2158	4948	0
H-84	104296	6532	52113402	17627202	21988	93934	28048706	40.89	1449	765	3489	10462	0
H-94	76852	8545	46004685	20963430	21466	106437	29836747	40.46	1748	884	3904	10053	0
H-380	88383	8069	50143507	21206337	21592	201711	30379429	39.73	1832	867	3522	9768	0
S-77	17739	159	5191068	317970	1245	18875	1018700	40.25	744	592	386	775	0
S-81	77087	9127	47894129	23095093	21542	60535	31566111	39.8	1958	943	3800	9725	0
S-82	75961	5415	36288587	11200434	16970	29737	18991490	41.29	1236	732	3878	9013	0

Table S5

CF-76

Clinically Tested Antibiotcs	Clinical Results: 26.06.2012	Genomic Results: 04:07.2012
Pip-Tazobactam	Sensitive	No resistance detected
Caftazidim	Sensitive	No resistance detected
cefepime	Sensitive	No resistance detected
Aztreonam	Sensitive	No resistance detected
Imipenem	Sensitive	No resistance detected
Meropenem	Sensitive	No resistance detected
Amikacin	Sensitive	No resistance detected
Netilmicin	Sensitive	No resistance detected
Tobramycin	Sensitive	No resistance detected
Ciprofloxacin	Sensitive	No resistance detected
Colistin	Sensitive	No resistance detected

CF-00

Clinically Tested Antibiotics	Clinical Results: 14.03.2013	Genomic Results: 14.03.2013
Ampicillin	Sensitive	No resistance detected
Amoxicillin-Calv.	Sensitive	No resistance detected
Cefuroxim	Sensitive	No resistance detected
Ceftriaxon	Sensitive	No resistance detected
Meropenem	Sensitive	No resistance detected
Ciprofloxacin	Sensitive	No resistance detected
Co-Trimoxazol	Sensitive	No resistance detected

CF-82

Clinically Tested Antibiotcs	Clinical Results: 29.04.2012	Genomic Results: 10.07.2012
Pip Tazobactam	Sensitive	No resistance detected
Ceftazidim	Sensitive	No resistance detected
Cefepime	Sensitive	No resistance detected
Aztreonam	Sensitive	No resistance detected
Imipenem	Sensitive	No resistance detected
Meropenem	Sensitive	No resistance detected
Amikacin	Sensitive	No resistance detected
Netilmicin	Sensitive	No resistance detected
Tobramycin	Sensitive	No resistance detected
Ciprofloxacin	Intermediate	Resistant
Colistin	Sensitive	Resistant

CF-94

Clinically Tested Antibiotics	Clinical Results: 06.08.2012	Genomic results:08.08.2012
Penicillin G	Resistant	No resistance detected
Ampicillin	Resistant	No resistance detected
Oxacillin	Sensitive	No resistance detected
Amoxicillin-Clav.	Sensitive	No resistance detected
Cefazolin	Sensitive	No resistance detected
Cefamandol	Sensitive	No resistance detected
Cefuroxim	Sensitive	No resistance detected
Imipenem	Sensitive	No resistance detected
Meropenem	Sensitive	No resistance detected
Rifampicin	Sensitive	No resistance detected
Ciprofloxacin	Sensitive	No resistance detected
Co-Trimoxazol	Sensitive	No resistance detected
Clindamycin	Sensitive	No resistance detected
Azithromycin	Sensitive	No resistance detected
Clarithromycin	Sensitive	No resistance detected
Erythromycin	Sensitive	No resistance detected
Tetracyclin	Sensitive	No resistance detected
Fusidinsäure	Sensitive	No resistance detected

CF-99

Clincally Tested Antibiotics	Clinical Results: 15.05.2013	Genomic Results: 15.05.2013
Penicillin G	Resistant	No resistance detected
Ampicillin	Resistant	No resistance detected
Oxacillin	Sensitive	No resistance detected
Amoxicillin-Clav.	Sensitive	No resistance detected
Pip-Tazobactam	Sensitive	No resistance detected
Cefamandol	Sensitive	No resistance detected
Cefuroxim	Sensitive	No resistance detected
Imipenem	Sensitive	No resistance detected
Meropenem	Sensitive	No resistance detected
Gentamicin	Sensitive	No resistance detected
Rifampicin	Sensitive	No resistance detected
Ciprofloxacin	Resistant	No resistance detected
levofloxacin	Sensitive	No resistance detected
Co-Trimoxazol	Sensitive	No resistance detected
Clindamycin	Sensitive	No resistance detected
Azithromycin	Sensitive	No resistance detected
Clarithromycin	Sensitive	No resistance detected
Erythromycin	Sensitive	No resistance detected
Tetracyclin	Sensitive	No resistance detected
Fusidinsäure	Sensitive	No resistance detected

CF-99_2

Clinically Tested Antibiotics	Clinical Results:23.02.2012	Genomic results:23.02.2012
Penicillin G	Resistant	No resistance detected
Ampicillin	Resistant	No resistance detected
Oxacillin	Sensitive	No resistance detected
Amoxicillin-Clav.	Sensitive	No resistance detected
Cefazolin	Sensitive	No resistance detected
Cefamandol	Sensitive	No resistance detected
Cefuroxim	Sensitive	No resistance detected
Imipenem	Sensitive	No resistance detected
Meropenem	Sensitive	No resistance detected
Gentamicin	Sensitive	No resistance detected
Tobramycin	Sensitive	No resistance detected
Rifampicin	Sensitive	No resistance detected
Ciprofloxacin	Sensitive	No resistance detected
Co-Trimoxazol	Sensitive	No resistance detected
Clindamycin	Sensitive	No resistance detected
Azithromycin	Sensitive	No resistance detected
Clarithromycin	Sensitive	No resistance detected
Erythromycin	Sensitive	No resistance detected
Tetracyclin	Sensitive	No resistance detected
Fusidinsäure	Sensitive	No resistance detected
